# Cuttlefish Ink-Derived Melanin Nanoparticles Enabling NIR-Responsive Electrospun Nanofibrous Mats for On-Demand Selective Antibacterial Disinfection of Orthodontic Braces

**DOI:** 10.1021/acsami.6c05032

**Published:** 2026-06-01

**Authors:** Magdalena Bartolewska, Daniel Rybak, Alicja Kosik-Kozioł, Piotr Jenczyk, Michał Pruchniewski, Dariusz Jarząbek, Massimiliano Lanzi, Filippo Pierini

**Affiliations:** † Department of Biosystems and Soft Matter, 86911 Institute of Fundamental Technological Research, Polish Academy of Sciences, Warsaw 02-106, Poland; ‡ Department of Mechanics of Materials, Institute of Fundamental Technological Research, Polish Academy of Sciences, Warsaw 02-106, Poland; § Department of Nanobiotechnology, Institute of Biology, 49561 Warsaw University of Life Sciences, Warsaw 02-786, Poland; ¶ Department of Industrial Chemistry “Toso Montanari”, University of Bologna, Viale Risorgimento 4, Bologna 40129, Italy

**Keywords:** cuttlefish ink, melanin nanoparticles, nanofibrous mats, photothermal, antibacterial, orthodontic braces

## Abstract

Fixed orthodontic appliances facilitate bacterial accumulation on brackets and wires, increasing the risk of enamel demineralization and periodontal inflammation. To address this challenge, near-infrared (NIR) responsive nanofibrous mats were developed for on-demand antibacterial disinfection of orthodontic brackets by incorporating cuttlefish ink-derived melanin nanoparticles (MNPs) into a poly­(vinyl alcohol)/poly­(ethylene oxide) (PVA/PEO) matrix. The incorporation of MNPs improved physicochemical properties, including enhanced thermal stability (∼77 °C increase in decomposition temperature), increased swelling capacity (∼2-fold compared with melanin-free fibers), and improved wettability. After thermal cross-linking, the fibrous network remained structurally stable in aqueous conditions with morphology preserved for up to 1 month and low melanin loss. Strong antioxidant performance was observed, reaching ∼60% 2,2-diphenyl-1-picrylhydrazyl (DPPH) radical-scavenging after 10 h. *In vitro* evaluation using L929 fibroblasts confirmed good cytocompatibility, supporting adhesion, viability, and proliferation. Under NIR irradiation at 808 nm (1.5 W/cm^2^, 10 min), efficient photothermal heating was achieved under wet conditions (∼55–60 °C) with stable performance across repeated cycles. Antibacterial efficacy was demonstrated, reducing *Escherichia coli* survival to 0.55% and disinfecting bacteria-contaminated bracket surfaces, while only minor inhibition of *Lactobacillus acidophilus* was detected. Overall, a biocompatible, marine-derived, and sustainable nanofibrous mat are presented for on-demand orthodontic disinfection.

## Graphical abstract

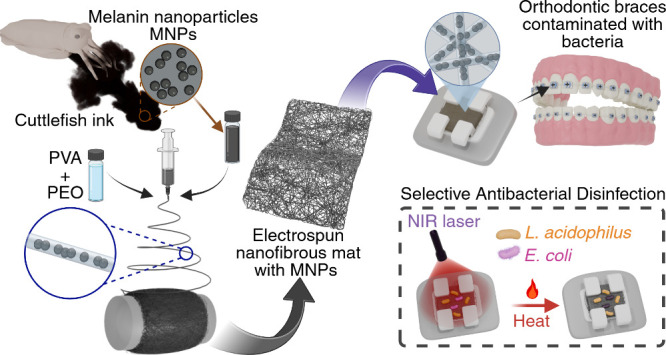

## 1 Introduction

Fixed orthodontic appliances remain a mainstay for correcting malocclusion; however, brackets, bands, and wires create retentive areas that are difficult to clean and favor bacterial colonization.^1^ Orthodontic treatment has been associated with increased levels of microorganisms such as *Streptococcus mutans* (*S. mutans*), *Lactobacillus acidophilus* (*L. acidophilus*), as well as other species, including *Escherichia coli* (*E. coli*), which may be detected in oral biofilms.^2,3^ The proliferation of these microorganisms increases the risk of enamel demineralization and periodontal inflammation.^1,4^ Controlling bacterial growth is a primary step in preventing enamel demineralization during orthodontic treatment.^4,5^ Accordingly, several preventive strategies aim to limit microbial accumulation, including the use of antibacterial mouthrinses and toothpastes.^5^ However, the effectiveness of these measures depends largely on consistent patient compliance.^5^ Additionally, some widely used mouthrinses, such as chlorhexidine, are associated with adverse effects including tooth discoloration, taste disturbance, and mucosal irritation.^6^

To address these limitations, novel strategies need to be developed. Antibacterial bracket coatings incorporating nanochitosan, silver, or zinc oxide nanoparticles have been investigated.^7,8^ Recently, Barylyak et al. prepared an orthodontic bracket adhesive doped with sulfur-modified TiO_2_ nanoparticles, demonstrating enhanced antibacterial activity while maintaining adequate mechanical and bonding properties.^9^ A recent review by Wang et al. also highlighted growing interest in the use of photothermal antibacterial therapy for oral applications.^10^ Photothermal therapy (PTT) has emerged as a promising antimicrobial strategy that eliminates bacteria and disrupts biofilms by generating localized heat.^11^ This effect is achieved using photothermal agents (PTA), materials that absorb light energy and convert it into heat, damaging bacterial membranes.^11^ Near-infrared (NIR) light is most commonly used for photothermal activation because it penetrates tissues more effectively than visible or UV light, enabling efficient heating at the target site.^12^

Importantly, selecting an appropriate PTA is critical to minimize toxicity and ensure biocompatibility. Natural materials are particularly attractive in this regard, with melanin emerging as a promising candidate due to its rapid and efficient photothermal conversion.^13^ Melanin occurs widely in nature, including in skin,^14^ hair,^15^ bacteria,^16^ fungi,^17^ plants and seeds,^18^ mussel shells,^19^ and the ink of squid and cuttlefish.^20,21^ Cuttlefish ink is especially interesting because it contains melanin in the form of small, spherical nanoparticles (∼100 nm),^22^ which can be readily incorporated into various materials for biomedical applications. The use of cuttlefish-derived nanoparticles in nanomaterials has been extensively investigated,^23–26^ whereas their incorporation into electrospun nanofibrous mats is relatively recent. The combination of nanofibrous scaffolds with a photothermal agent highlights the complementary roles of these material classes. Photoresponsive nanoparticles provide light-induced functionality, while electrospun nanofibrous mats offer a porous, mechanically stable matrix.

Electrospinning is a promising method for fabricating melanin-containing nanofibrous mats. Melanin nanoparticles (MNPs) cannot be electrospun on their own, so a carrier polymer is required. Water-processable polymers such as poly­(vinyl alcohol) (PVA) and poly­(ethylene oxide) (PEO) are particularly attractive, as they can be electrospun from aqueous solutions, avoiding toxic organic solvents. For example, Srisuk et al. incorporated cuttlefish-derived melanin into electrospun PVA nanofibers as an electrically active scaffold for skeletal muscle tissue engineering.^27^ Similarly, Bayrak et al. reported that incorporating squid ink-derived melanin into PVA nanofibrous mats enhanced wound closure under UV-A irradiation.^28^ More recently, Corsini et al. used cuttlefish ink as a photothermal agent in silk fibroin nanofiber membranes for laser-assisted tissue welding for soft tissue repair.^29^ To the best of our knowledge, no previous studies have reported the use of cuttlefish-derived melanin embedded in electrospun nanofibrous mats for NIR-mediated photothermal bacterial eradication.

This study aimed to design and evaluate NIR-responsive antibacterial nanofibrous mats for application on orthodontic brackets. A nanofibrous platform based on PVA/PEO incorporating melanin nanoparticles (MNPs) was developed, and the cross-linking of the electrospun nanofibrous mats was investigated. The chemical composition, morphology, thermal behavior, and mechanical properties of the nanofibrous mats were characterized to confirm the successful integration of MNPs into the PVA/PEO nanofibers. The photothermal performance of the electrospun nanofibrous mat under NIR irradiation was then evaluated. *In vitro* assays were conducted to assess the biocompatibility of nanofibers containing MNPs and to support their potential for biomedical applications, particularly for orthodontic bracket disinfection. The antibacterial efficacy of the nanofibrous mat was also examined against *E. coli* and *L. acidophilus* on orthodontic brackets. Overall, incorporating stimuli-responsive disinfection into orthodontic biomaterials offers a promising strategy to reduce bacterial colonization during orthodontic treatment. This approach may help prevent periodontal disease, limit enamel damage, and improve oral health outcomes. The study also highlights the valorization of marine-derived melanin from cuttlefish ink within a nanofibrous network, expanding its potential as a photothermal material for antibacterial applications.

## 2 Results and Discussion

The focus of this study was the development of a nanofibrous mat incorporating MNPs derived from cuttlefish ink, which was achieved through electrospinning into a PVA/PEO polymer matrix, as illustrated in Scheme 1a. Owing to the strong NIR absorption of melanin, the resulting nanofibrous mats exhibited significant photothermal properties. Upon NIR irradiation, localized heat generation occurred, leading to thermal inactivation and killing of bacteria on the bracket surface. As schematically depicted in Scheme 1b, the photothermally induced disinfection effectively eradicated the Gram-negative model organism *E. coli*, while *L. acidophilus* remained unaffected, demonstrating the selective antibacterial effect of the MNP-based nanofibrous mats.

**Scheme 1:**
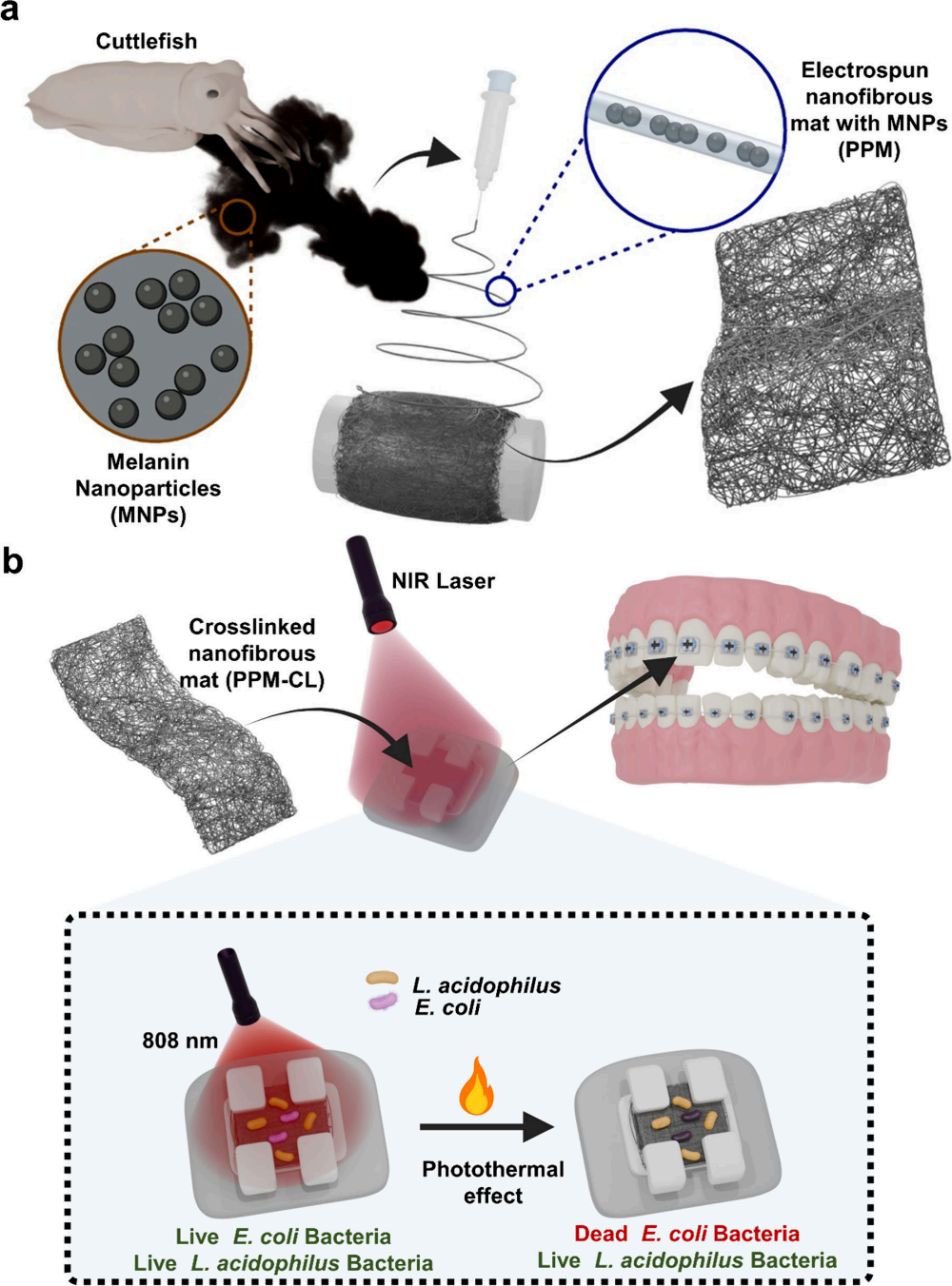
Schematic Illustration of the Fabrication and Antibacterial Application of the Melanin Nanoparticles (MNPs) Incorporated into a Nanofibrous Mat

a) Preparation of MNP-loaded PVA/PEO nanofibrous mat (PPM) via electrospinning. b) Photothermal disinfection mechanism of orthodontic brackets coated with the nanofibrous mat, where NIR light irradiation induces localized heat generation, leading to thermal inactivation of *E. coli* and a slight reduction in *L. acidophilus*.

### 2.1 Melanin Nanoparticles

MNPs were purified from cuttlefish ink by multiple centrifugal washing steps with deionized water to remove residual salts, followed by freeze-drying, yielding a black powder (Figure S1). The morphology of the MNPs was characterized by scanning electron microscopy (SEM) (Figure 1a) and scanning transmission electron microscopy (STEM) (Figure 1b), revealing predominantly spherical particles with a relatively narrow size distribution and an average diameter of approximately 130.01 ± 17.01 nm. The hydrodynamic diameter measured by dynamic light scattering (DLS) is approximately 227.97 ± 1.63 nm, with a polydispersity index of ∼0.1 (Figure 1c). The MNPs suspension also exhibited a negative zeta potential of −38.87 ± 0.71 mV, attributed to surface polyphenolic groups that electrostatically stabilize the nanoparticles in aqueous media.^25^

**Figure 1:**
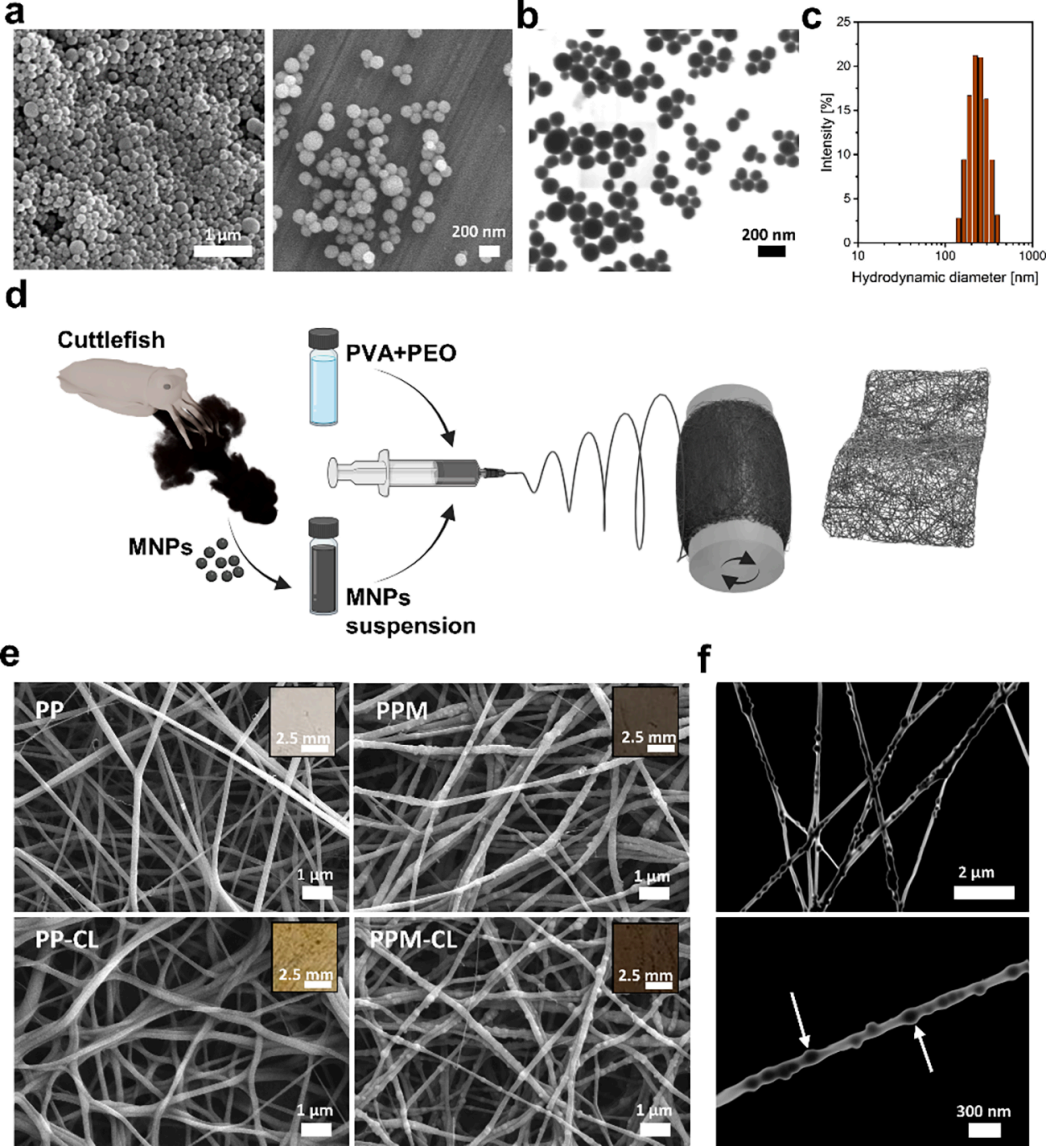
Melanin nanoparticle characterization and incorporation into electrospun nanofibrous mat. a) FE-SEM images of melanin nanoparticle (MNP) powder. b) STEM image of the electrosprayed MNP suspension. c) DLS size distribution of MNPs. d) Schematic illustration of MNP extraction from cuttlefish ink and subsequent incorporation into the electrospinning process. e) FE-SEM images showing the morphology of the electrospun nanofibrous mat before and after thermal cross-linking (-CL) with (PPM) and without MNPs (PP). Photograph of the nanofibrous mat with insets shown in the upper right corner. f) STEM image of cross-linked nanofibers containing MNPs (PPM-CL). White arrows highlight incorporated nanoparticles.

The purified melanin powder obtained from cuttlefish ink was further analyzed by attenuated total reflectance Fourier transform infrared (ATR-FTIR) spectroscopy to confirm its chemical structure (Figure S2). A peak signal in the range from 3670 to 2650 cm^–1^ is attributed to the O–H and N–H stretching vibrations of the carboxyl, phenolic, and amino functional groups. The small peak at 2940 cm^–1^ is from −CH stretching. A characteristic strong band at 1589 cm^–1^ (reported in most literature around 1620 cm^–1^) is attributed to bending vibrations of aromatic ring C=C, along with C=O stretching vibrations from carboxylic groups. Additionally, the peak at 1352 cm^–1^ corresponds to C–N stretching at the indole ring. These characteristic peaks were highly consistent with the natural MNPs reported in previous studies.^30^

### 2.2 Electrospun Nanofibrous Mats Embedded with MNPs

Electrospinning was employed to fabricate continuous PVA/PEO nanofibers and to achieve uniform incorporation of MNPs within the fibrous matrix, as schematically illustrated in Figure 1d. MNPs were first isolated from cuttlefish ink and subsequently dispersed in a DI water/ethanol mixture (50:50, v/v) at a concentration of 25 mg/mL to obtain a stable suspension. For electrospinning, the prepared solution was loaded into a 1 mL syringe fitted with a 22G needle. An 18 kV high-voltage source was applied to generate the polymer jet. The flow rate was set at 200 μL/h, and the nanofibers were collected on a rotating drum collector operating at 400 rpm to ensure uniform mat thickness and homogeneous fiber deposition.

Under these optimized conditions, uniform and bead-free PVA/PEO nanofibrous mats were successfully fabricated both without MNPs (PP) and with MNPs (PPM), as shown in Figure 1e. The mean fiber diameters were 153.47 ± 42.34 nm and 191.31 ± 39.88 nm, respectively. Incorporation of melanin nanoparticles increased the average fiber diameter.

Since both PVA and PEO are water-soluble polymers, thermal cross-linking was performed to maintain the fibrous architecture under aqueous conditions. The nanofibrous mats were heated at 150 °C for 2 h to promote physical cross-linking of PVA by increasing crystallinity and strengthening intermolecular hydrogen bonding between PVA chains. This treatment can also promote hydrogen bonding between carbonyl (C=O) groups and PVA hydroxyl (−OH) groups.^31^ Although PEO does not thermally cross-link under these conditions, it can form hydrogen bonds with PVA chains.^32^ Additionally, melanin nanoparticles contain catechol and quinone functional groups capable of forming hydrogen-bonding interactions, which may further stabilize the nanofibrous network.^33,34^ SEM analysis after cross-linking revealed morphological changes in the nanofibers (Figure 1e). In the PP cross-linked (PP-CL), a noticeable increase in the average fiber diameter (194.10 ± 61.97 nm) was observed after cross-linking. This increase may be attributed to partial fiber–fiber fusion, in which neighboring nanofibers became connected, forming thicker, merged structures rather than individual thin fibers.

In contrast, the PPM cross-linked (PPM-CL) nanofibers exhibited a reduced mean fiber diameter (137.38 ± 22.69 nm) while preserving their fibrous morphology after thermal treatment. The decrease in diameter improved the visibility of the spherical features associated with the incorporated MNPs, making the nanoparticles more distinguishable. Unlike PP-CL, where partial fiber–fiber fusion could be observed after cross-linking, PPM-CL largely maintained a single fiber structure. An additional confirmation of successful melanin incorporation was the visible color change of the nanofibrous mats. As shown in Figure 1e (top-right insets), photographic images of the electrospun nanofibrous mat before and after thermal cross-linking reveal a distinct difference in appearance. The electrospun mats exhibited a clear color transition from white to dark brown, consistent with the presence of melanin nanoparticles. The control PP nanofibrous mat remained white after electrospinning but displayed a slight yellowish tint following thermal cross-linking.^31^ STEM analysis was further performed to confirm the incorporation of nanoparticles within the cross-linked nanofibers (Figure 1f). Electron-dense MNPs were clearly observed embedded within the fiber matrix and are highlighted with white arrows in the corresponding micrographs. In contrast, no nanoparticle-like structures were detected in the control PP sample (Figure S3), confirming that the observed particles in the PPM samples correspond to successfully incorporated MNPs.

### 2.3 Characterization of Nanofibrous Mats

To investigate the incorporation of MNPs into the polymer nanofibrous mat, ATR-FTIR analysis was performed. As presented in Figure S4, hydrogen bond formation between PVA/PEO and melanin, which would typically be indicated by a shift or broadening of the −OH stretching band, is not clearly confirmed in our results, despite such effects being reported in the literature.^34,35^ A possible explanation is that strong intermolecular hydrogen bonding already occurs between PVA and PEO chains. In PVA/PEO blends, hydrogen bonds can form between the hydroxyl (−OH) groups of PVA and the ether oxygen atoms of PEO, leading to a shift in the −OH stretching band.^32,36^ However, the lower intensity of the O–H band observed in PPM is likely due, at least in part, to hydrogen bond formation between PVA and melanin. For both PP and PPM, there is a peak that appears at 1718 cm^–1^. That could be due to the stretching of the C=O and C–O from the acetate group remaining from partially hydrolyzed PVA.^32^ In the PPM spectra, bands at 1378, 1537 cm^–1^ and 1616 cm^–1^ are also observed and are consistent with indole ring and indolic structures, with contributions of bending vibrations of the aromatic ring C=C and C=N bond.^27,37^

In Figure 2a, ATR-FTIR spectra of the MNPs powder and the cross-linked nanofibrous mats are shown. After thermal treatment, hydrogen-bond formation became more pronounced, leading to a decrease in band intensity and a shift toward lower wavenumbers (from 3320 cm^–1^ to 3302 cm^–1^). This suggests that elevated temperature promotes hydrogen bond formation. The peak at 1718 cm^–1^ remained after thermal cross-linking for PPM-CL, which may be attributed to C=O stretching of ester groups, suggesting that carboxylic acid groups from eumelanin and hydroxyl groups from PVA could react during thermal treatment via esterification (peak around 1724 cm^–1^ highlighted with a black arrow).^35^

**Figure 2:**
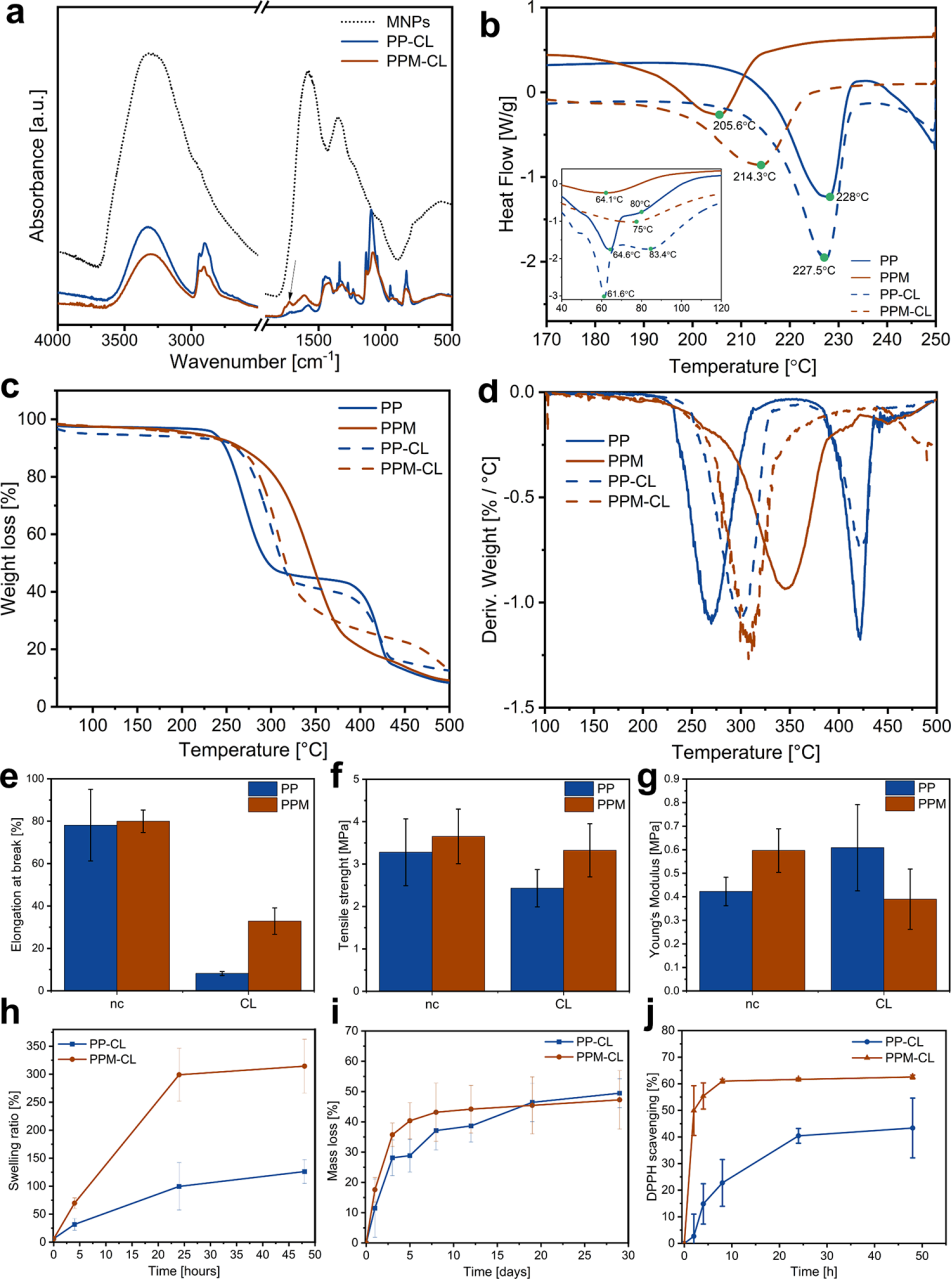
Physicochemical characterization: a) ATR-FTIR spectra of the nanofibrous mats and MNPs, b) first DSC heating scans, c) TGA thermograms, and d) first derivative of the TGA curves. Mechanical properties of nanofibrous mats e) elongation at break, f) tensile strength of the nanofibrous mats, and g) Young’s modulus of the nanofibrous mats. h) Swelling behavior of the nanofibrous mats. i) Degradation profile of the nanofibrous mats. j) Antioxidant properties of the nanofibrous mats.

The thermal behavior of the prepared nanofibrous mats and their phase transition temperatures were analyzed by differential scanning calorimetry (DSC) to better understand their structure–property relationships (Figure 2b). PVA and PEO exhibit different melting and glass transition temperatures. For PVA, the glass transition reported in the literature occurs at approximately 74 °C,^32^ and in Figure 2b it is observed near 80 °C. Additionally, a peak at 64.6 °C was observed, corresponding to the melting temperature of PEO, reported in the literature to be around 65 °C.^32^ This transition is characterized by a high enthalpy (138.3 J/g), indicating that the observed signal arises from both PEO melting and the PVA glass transition. However, the enthalpy and peak shape change after melanin incorporation. The PPM sample shows a reduced enthalpy of 57.81 J/g, indicating interactions between the polymer components. This is further supported by the decrease in melting temperature from 228 to 205.6 °C after melanin addition, suggesting reduced crystalline order due to disruption of polymer chain packing. This effect may result from interactions between functional groups of the polymers and melanin, consistent with results reported by Wang et al.^34^

Thermal cross-linking also influences the melting temperature of both PP-CL and PPM-CL nanofibrous mats. For PP-CL fibers, cross-linking resulted in only a minimal decrease in Tm of approximately 0.5 °C. Thermal treatment (annealing) can increase crystallinity and promote chain rearrangement; however, at 150 °C, some easily cleavable polymer chains or macromolecular groups may undergo chain scission or partial decomposition, observed as yellowing (Figure 1e). This degradation could explain the slight decrease in melting temperature and is consistent with the findings of Miraftab et al.^38^

Figure 2c and 2d present the thermal degradation behavior of the PP and PPM nanofibrous mats. It has been previously reported that the thermal degradation of PVA/PEO blends proceeds through three stages. The first stage, occurring at approximately 70–80 °C, is associated with the loss of absorbed moisture.^32^ However, this stage is not clearly resolved in the present thermogravimetric analysis (TGA) curves. The second degradation stage, observed around 265 °C, is mainly due to PVA degradation. The third stage, occurring near 420 °C, corresponds to further decomposition of the polymer matrix. In contrast, PEO typically exhibits a single major degradation step beginning at approximately 379 °C, which is attributed to random chain scission of C–O bonds.^32^ The incorporation of melanin nanoparticles increases the decomposition temperature by ∼77 °C, indicating enhanced thermal stability. Similar increases in onset temperature have been reported for sepia melanin in PMMA,^39^ and for melanin PVA blends.^33^ This temperature increase was found to be attributed to melanin’s ability to scavenge free radicals generated from the unsaturated polymer chains and it exerts a blocking effect on the unzipping depolymerization mechanism. As a result, the polymer/melanin blends degraded predominantly via random chain scission at temperatures above 300 °C.^33,39^

Thermal cross-linking generally shifts the degradation process to higher temperatures than in non-cross-linked samples, as observed for the PP-CL nanofibrous mat. This shift results from partial thermal degradation occurring earlier at 150 °C.^40^ However, for the PPM-CL nanofibrous mat, the decomposition temperature decreases slightly, suggesting that the combined effects of melanin incorporation and cross-linking modify the degradation mechanism. A similar trend was reported by Eom et al. for PVA/melanin composites, where the degradation onset temperature decreased after melanin addition; they attributed this effect to efficient heat spreading at the surfaces of melanin nanoparticles.^41^ Additionally, a significant change in the shape of the degradation peak is observed for the PPM and PPM-CL nanofibrous mats. As shown in Figure 2d, the degradation behavior is characterized by a single dominant peak at higher temperature.

### 2.4 Mechanical Properties

Natural eumelanin contains reactive functional groups (−OH, -NH, −COOH, and catechols), enabling strong interfacial interactions and effective cross-linking with a wide range of polymers to improve mechanical performance.^42^ For example, incorporating melanin into polymer matrices has been shown to increase Young’s modulus, tensile strength, and elongation at break.^34,42–44^ To evaluate the effect of melanin modification, the mechanical properties of the nanofibrous mats were measured with and without MNPs, before and after thermal cross-linking (-CL). The representative stress–strain curves of the nanofibrous mats (Figure S5) provide an overview of their mechanical behavior before and after thermal cross-linking. Non-cross-linked samples (PP and PPM) demonstrate characteristic flexible behavior with significant extension, while cross-linked samples exhibit reduced elongation and a more brittle reaction. The inset images further confirm these differences, revealing more stretched and thinned nanofibrous mats with irregular fracture edges before cross-linking, compared to more uniform and aligned fracture surfaces after cross-linking. As shown in Figure 2e, the elongation at break of the non-cross-linked samples did not change significantly upon incorporation of MNPs. However, following thermal cross-linking, a 70% decrease in elongation at break was observed, indicating reduced flexibility of the nanofibrous structure. Notably, the presence of MNPs partially preserved the elongation capability after cross-linking to 32%, suggesting that melanin contributes to maintaining a certain degree of flexibility within the thermally stabilized network.

The tensile strength of the non-cross-linked nanofibrous mats was slightly improved upon incorporation of MNPs (Figure 2f). After thermal cross-linking, a slight decrease in tensile strength was observed compared with the corresponding non-cross-linked samples. Nevertheless, melanin incorporation improved the tensile strength of both cross-linked and non-cross-linked nanofibrous mats relative to their respective controls.

Young’s modulus values are presented in Figure 2g. In the non-cross-linked samples, incorporation of MNPs increased the modulus from approximately 0.42 to 0.60 MPa, indicating enhanced stiffness. For the cross-linked PP sample, the Young’s modulus increased from 0.42 to 0.61 MPa after thermal treatment. In contrast, the Young’s modulus of the cross-linked PPM sample decreased to 0.39 MPa compared with its non-cross-linked counterpart. This reduction in mechanical performance after thermal cross-linking is primarily attributed to structural rearrangements within the nanofibrous mat. Heat treatment can alter fiber morphology, interfiber bonding, and polymer chain organization, leading to changes in stress distribution under tensile loading. In this system, PVA undergoes cross-linking at the applied temperature, while PEO remains non-cross-linked and may partially melt, which can lead to localized structural heterogeneity and reduced reinforcement within the fiber network.

The decrease in Young’s modulus differs from that previously reported for PVA/melanin systems, where thermal treatment at 160 °C resulted in an increased Young’s modulus for PVA/melanin compared to pure PVA.^34^ Similarly, Eom et al. reported that treatment at elevated temperatures did not reduce the Young’s modulus of PVA/melanin composites.^41^ However, these studies are based on bulk solvent-cast films rather than nanofibrous mats, which is a fundamental geometric difference. In addition to the role of PEO, the presence of MNPs may further interfere with the cross-linking process. Eom et al. reported that MNPs efficiently spread heat through the polymer matrix due to the thermal carriers present in their conjugated structure, and that this heat-spreading effect lowers the onset of PVA degradation in MNP/PVA composites.^41^ Given the comparable size of the MNPs and the nanofiber diameter, the localized heat spreading from each MNP surface may degrade the surrounding PVA and reduce the local cross-linking density, altering the mechanical properties and contributing to the observed decrease in Young’s modulus and tensile strength of PPM-CL.

### 2.5 Swelling and Degradation

Eumelanin is hydrophilic and has a strong capacity to absorb atmospheric moisture.^45^ Consistent with this, incorporating MNPs into the nanofibrous mats markedly increased swelling. The PPM-CL showed approximately double the swelling compared with PP-CL, as shown in Figure 2h. A similar increase in swelling of nanofibrous mats was reported by Corsini et al. when melanin was added.^29^ The contact angle results support the same trend, with PPM exhibiting a lower contact angle than PP (decreasing from 25.4 ± 0.5° to 17.6 ± 1.6°). Moreover, the remaining droplet after the measurement increased in diameter (from 1.0 to 1.3 cm), confirming that MNPs addition improves wettability. However, some studies have reported the opposite trend, with contact angle increasing after melanin addition.^28^

Since the intended application of the nanofibrous mats involves prolonged exposure to moisture, degradation behavior and potential material loss are important factors. Therefore, the nanofibrous mat degradation was evaluated over several weeks. Mass loss was calculated after maximum swelling had been achieved, and the degradation profile of mass changes over time is presented in Figure S6. At longer time points, both PP-CL and PPM-CL exhibit a similar total mass loss (approximately 40–50%), which is primarily attributed to the dissolution of PEO from the nanofibrous mat. However, on the first day, PPM-CL shows a slightly higher mass loss, which may be related to the release of MNPs (Figure 2i). Although thermal treatment at 150 °C promotes physical cross-linking of PVA by increasing crystallinity and strengthening intermolecular hydrogen bonding, PEO does not undergo cross-linking under these conditions and remains water-soluble. Consequently, upon immersion in PBS, PEO gradually dissolves and is released from the fiber matrix, accounting for the majority of the observed mass loss. The release of MNPs remained low throughout the study, reaching approximately 0.6 mg/mL after 31 days (Figure S7), and should not raise safety concerns, as the nanoparticles are derived from cuttlefish ink, a common food additive.^46^ Moreover, previous studies have reported beneficial effects associated with oral supplementation of natural melanin. Notably, acute oral toxicity assessment of *Sepia officinalis* ink extract, conducted in accordance with OECD Guideline 420, demonstrated no observable toxicity in rats at doses up to 2000 mg/kg.^47^ Additionally, several *in vivo* studies have tested oral administration of melanin at doses ranging from 75 to 480 mg/kg, consistently reporting anti-inflammatory rather than pro-inflammatory effects, including down-regulation of TNF-α, IL-1β, and IFN-γ and up-regulation of IL-10.^48,49^ Therefore, the small loss of melanin from the nanofibrous mats is not expected to pose a safety concern. Furthermore, SEM images of the nanofibrous mats after incubation at each time point demonstrate that the cross-linked structure is well maintained, even after one month (Figure S8). This indicates that PP-CL and PPM-CL are well cross-linked.

### 2.6 Antioxidant Properties

Melanin is well-known for its strong antioxidant activity, which represents one of its key biological functions. Owing to its ability to both donate and accept electrons, melanin can participate in single-electron transfer reactions that enable the neutralization of free radicals and peroxide species.^13,50^ The antioxidant potential of melanin has been extensively investigated in marine organisms, particularly in cephalopods, where it plays an important protective role against oxidative stress.^51^

To evaluate its antioxidant properties, a standard assay for free-radical scavenging activity was performed using the stable 2,2-diphenyl-1-picrylhydrazyl (DPPH) radical.^52^ As shown in Figure 2j, PPM-CL exhibits significantly higher DPPH radical-scavenging activity than PP-CL, reaching a maximum of ∼60% after 10 h of incubation. A small degree of radical-scavenging activity is also observed for PP-CL, although the inhibition is markedly lower than that of PPM-CL. The scavenging level obtained with PPM-CL is comparable to that previously reported for melanin-loaded electrospun nanofibrous mats. For example, melanin incorporated into PAN electrospun nanofibrous mats showed DPPH scavenging rates of 48%, while neat PAN nanofibers exhibited no antioxidant activity.^53^ It is important to note that melanin from different biological sources can exhibit different DPPH radical-scavenging efficiencies, because its chemical composition and functional groups vary depending on origin and extraction conditions.^13,54^ In addition, melanin’s antioxidant activity has been demonstrated *in vitro*, including cellular models in which melanin reduced oxidative stress markers.^51^

### 2.7 Photothermal Characterization

MNPs are recognized for their high photothermal conversion efficiency and broad optical absorption spanning ultraviolet to NIR wavelengths. A key characteristic of the photophysical and photochemical processes involved in eumelanin excitation and relaxation is their ultrafast conversion of absorbed radiation into heat, which occurs within 1 ns.^55^ Consequently, natural melanin nanoparticles have increasingly attracted attention for photothermal applications.^13^

To evaluate the photothermal effect of MNPs addition to nanofibers, an 808 nm NIR laser was used for sample irradiation, and temperature–time profiles were recorded using a thermal camera, as shown in Figure 3. Under dry conditions, rapid heating of the nanofibrous mats was observed during laser irradiation. Even at low power densities, temperatures reached approximately 65–70 °C, while at higher power (2 W/cm^2^) temperatures approached ∼126 °C (Figure 3a). However, in orthodontic applications, the nanofibrous mats are expected to function primarily in a moist environment. Therefore, measurements shown in Figure 3b were performed with nanofibrous mats immersed in 100 μL of water to simulate wet conditions. Under these conditions, the temperature increase was significantly reduced, with the maximum temperature decreasing from ∼126 °C to approximately 66.5 °C at 2 W/cm^2^. For photothermal inactivation and eradication of bacteria, temperatures in the range of ∼55–60 °C have been reported to be sufficient.^56^ Considering the potential adverse effects of excessive irradiation on surrounding tissues, an irradiance of 1.5 W/cm^2^ was selected as an optimal and safe operating condition.^57^ As indicated in Figure 3b by the red dashed line marking 60 °C, this threshold corresponds to the power level required for effective bacterial eradication. The investigation revealed a rapid temperature increase that was directly proportional to the applied laser power density (Figure 3c). A strong linear correlation was observed between the temperature rise and the laser intensity. Notably, this relationship was more linear under dry conditions (*R* ^2^ = 0.997), whereas a reduced linearity was observed in the wet state (*R* ^2^ = 0.951). Additionally, after four heating–cooling cycles, the highest temperatures of PPM-CL did not decrease (Figure 3d). These results indicate that PPM-CL maintains its photothermal performance over repeated irradiation cycles, highlighting its potential as a material for disinfecting orthodontic brackets.

**Figure 3:**
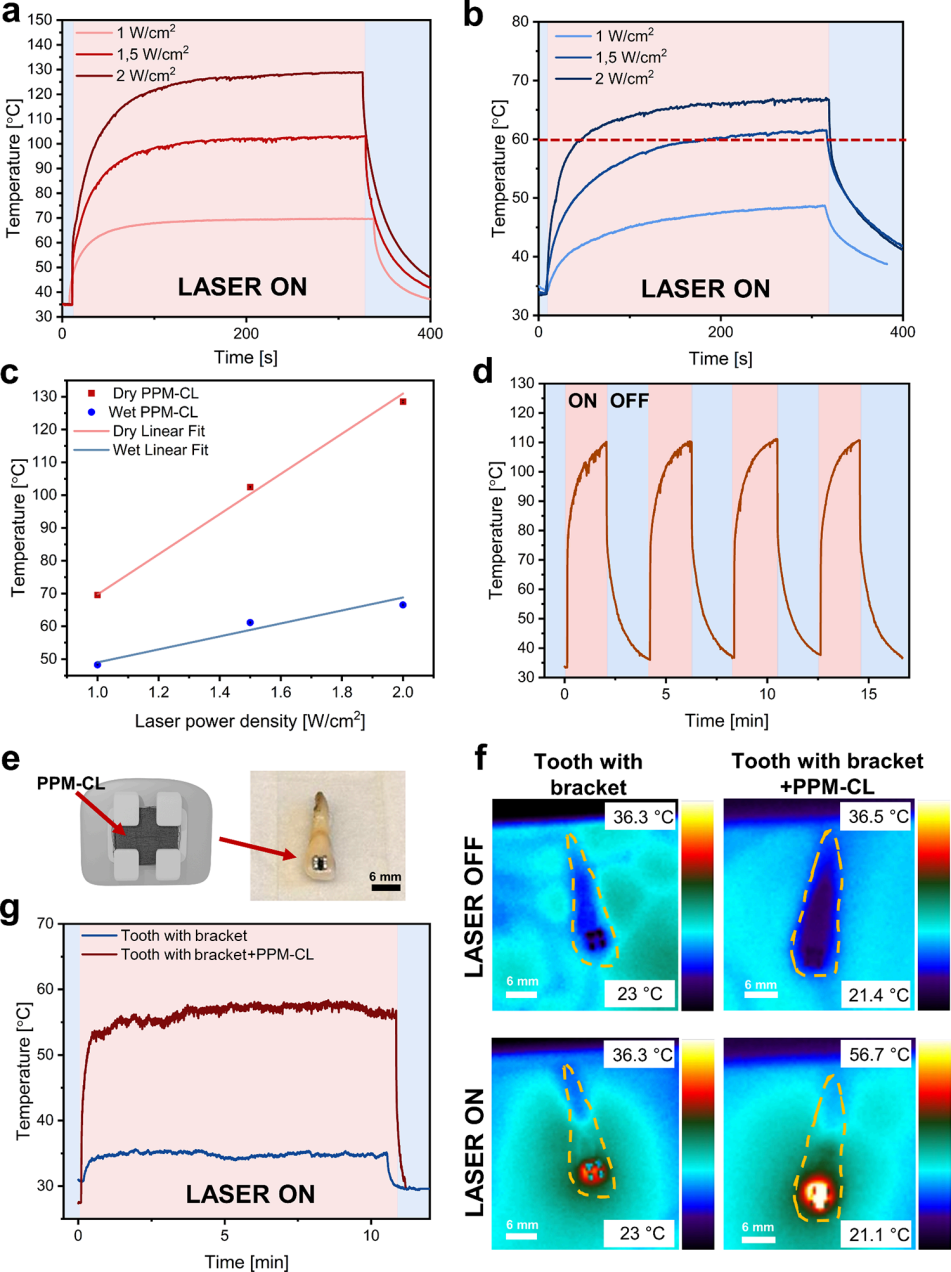
Photothermal properties of nanofibrous mats containing MNPs. Graphs showing the temperature–time profiles of PPM-CL at different laser power densities (1–2 W/cm^2^) in the a) dry state; b) wet state. c) Linear plot of laser power and temperature dependence for dry and wet states. d) Four NIR irradiation cycles demonstrate PPM-CL stability with temperatures reaching consistent levels across every cycle. e) Illustration of the experimental setup for photothermal testing on a tooth with an orthodontic bracket and nanofibrous mats. f) Thermal camera images showing a tooth with an orthodontic bracket, with and without PPM-CL, indicating the location of heat increase on the tooth, and g) photothermal response of orthodontic brackets with and without photothermal PPM-CL under irradiation at 1.5 W/cm^2^ for 10 min.

Teeth and the dental pulp can be sensitive to overheating.^58^ To evaluate the temperature rise produced during irradiation, the nanofibrous mats were mounted on an orthodontic bracket. The placement of the nanofibrous mats on the bracket is shown in Figure 3e, and the corresponding time–temperature profiles are presented in Figure 3g. Temperature monitoring is important, as excessive heat transfer through enamel and dentin may increase intrapulpal temperature and lead to pulpal injury.^58^ The spatial distribution of heat on the tooth and bracket under laser irradiation is presented in Figure 3f, where the tooth region is indicated by a dashed yellow line. The results clearly demonstrate that, in the absence of PPM-CL, the tooth surface does not undergo significant heating. In contrast, for PPM-CL, heat generation is localized exclusively on the bracket.

In our setup, irradiation of the tooth bracket with nanofibrous mats resulted in a modest temperature increase to approximately 35 °C (Figure 3g). In contrast, when PPM nanofibrous mats were attached to the bracket and irradiated, the temperature measured at the nanofibrous mats increased substantially, reaching ∼55–60 °C (Figure 3g). Importantly, the elevated temperature was primarily observed at the nanofibrous mats, whereas the tooth temperature remained much lower. This difference is expected because the bracket and adhesive layer act as intermediate materials between the heated nanofibrous mats and the enamel surface, reducing direct heat transfer to the tooth. Ran et al. reported that a tooth surface temperature of approximately 54.6 °C remained below the dentin tolerance threshold and was therefore considered safe.^59^ In addition, exposure to 55 °C for a short period of time was considered safe for the oral mucosa.^57^

### 2.8 *In Vitro* Cell Biocompatibility Studies

Several studies have demonstrated melanin’s biocompatibility with cells, showing low or no cytotoxicity.^60,61^ To evaluate the biocompatibility of the nanofibrous mats and the influence of melanin on cell interactions, tests with L929 fibroblast cells were performed in accordance with ISO 10993–12:2021 and ISO 10993–5. Initially, biocompatibility was assessed using a direct cell-seeding assay on PP-CL and PPM-CL nanofibrous mats. As a control, cells were seeded on a glass slide. Cell proliferation was monitored over 7 days. As shown in Figure 4a, proliferation on the nanofibrous mats was only slightly lower on day 7 compared with the control, indicating that the materials are not cytotoxic and do not significantly inhibit cell growth.

**Figure 4:**
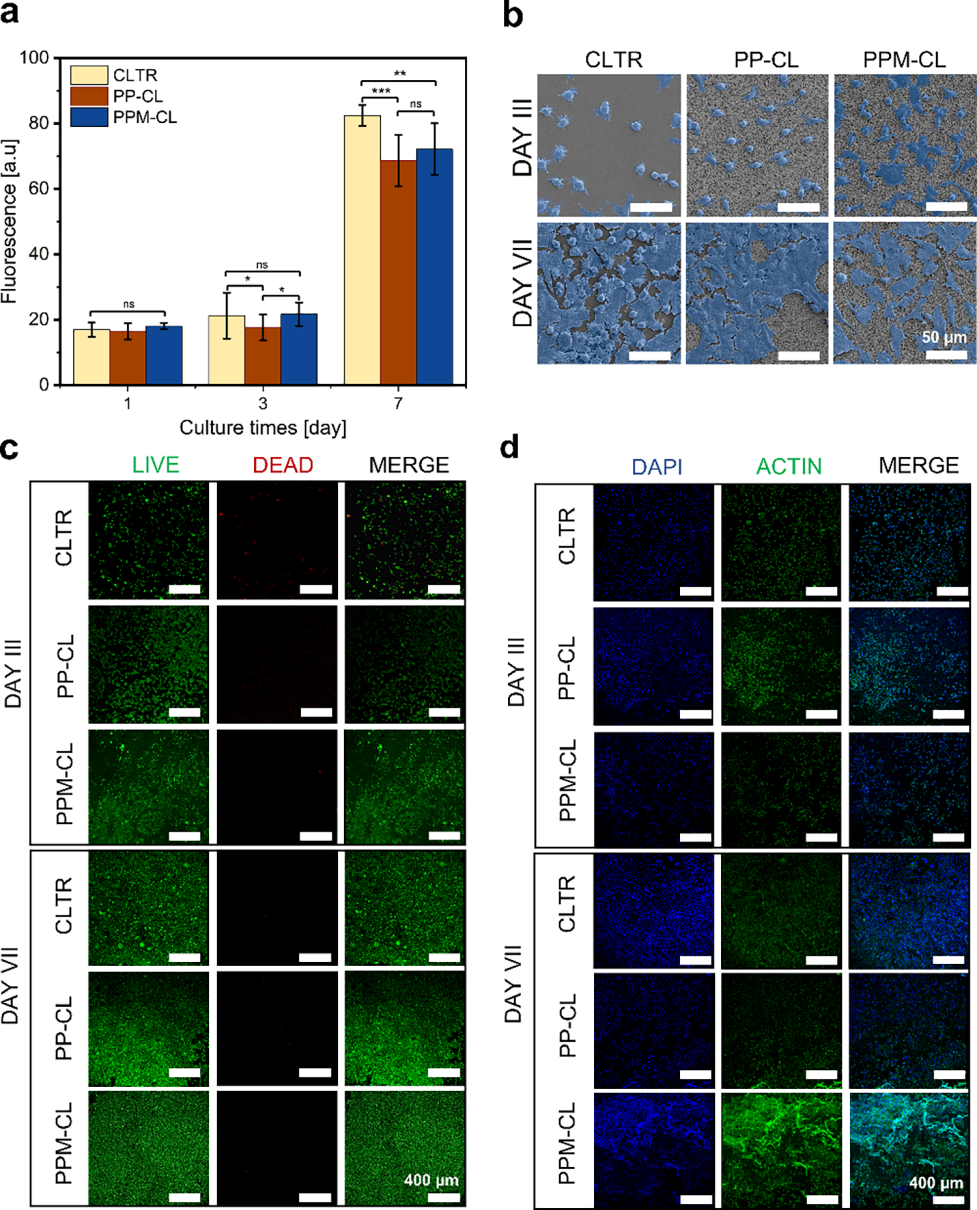
Biocompatibility of the nanofibrous mats. a) Proliferation of cells seeded on nanofibrous mats, assessed by fluorescence measurements on days 1, 3, and 7. b) False-colored SEM images of L929 cells (shown in blue) cultured on PP-CL and PPM-CL nanofibrous scaffolds and the control (CTRL) on glass slides on days 3 and 7, showing cell morphology and attachment to the scaffold. c) Confocal microscopy images of Live/Dead staining from indirect studies on days 3 and 7 (green, live cells; red, dead cells). d) Confocal images of L929 fibroblasts stained with DAPI for nuclei (blue) and actin for cytoskeleton (green) on days 3 and 7, used to assess cell health and morphology over time.

A similar result has been reported by Srisuk et al., who showed that cuttlefish-derived melanin incorporated into electrospun PVA nanofibrous mats enhanced the proliferation of C2C12 myoblasts compared to pure PVA fibers.^27^ In addition, cell morphology was examined by SEM on days 3 and 7 (Figure 4b). The images were false-colored, with cells shown in blue. As seen in Figure 4b, cells adhere well to both PP-CL and PPM-CL nanofibrous mats, exhibiting an elongated, spindle-like morphology. By day 7, the scaffold surface is largely covered by a continuous layer of cells.

To confirm cell viability on the nanofibrous mats, a Live/Dead assay was performed and is shown in Figure 4c. Images from the Live/Dead assay showed a predominance of viable (green) cells and only a small number of dead (red) cells on both day 3 and day 7, indicating good cytocompatibility of the materials. In addition, confocal microscopy was applied to analyze the morphology of the actin cytoskeleton and nuclei on days 3 and 7. L929 cells seeded on PP-CL and PPM-CL nanofibrous mats showed well-preserved intercellular connections. The nuclei appeared intact, as visualized by DAPI staining (Figure 4d).

### 2.9 NIR-Activated Antibacterial Properties

In the study by Perkowski et al., *E. coli* was detected in approximately 20% of young people aged 14–23 years who were treated with fixed orthodontic appliances.^3^ Based on these findings, a growth test was performed on *E. coli* under NIR light irradiation (1.5 W/cm^2^). *E. coli* was selected as a well-established Gram-negative model organism and because it has also been detected on orthodontic brackets. First, the irradiation time required to eradicate the bacteria was determined (Figure S9). An exposure of 5 min was insufficient, while 7 min resulted in improved bacterial reduction. After 10 min, most bacteria were eliminated, and after 15 min, none were detected. However, prolonged exposure may also be harmful to surrounding tissues; therefore, a 10 min irradiation time was chosen as the optimal and safe duration.

After 10 min of irradiation, bacterial survival was reduced to 0.55% (Figure 5a). As shown in Figure 5b, the representative agar plates demonstrate that only the PPM-CL + NIR treatment resulted in a clear reduction of bacterial growth, attributable to the temperature increase induced by photothermal heating. Similar photothermal eradication of *E. coli* has been reported by Qi et al. using graphene oxide-embedded polyvinylpyrrolidone electrospun nanofibrous mat under identical irradiation parameters (808 nm, 1.5 W/cm^2^, 10 min), confirming that these conditions are well established for photothermal antibacterial activity in nanofibrous mats.^62^ Although melanin may exhibit some antibacterial effects upon contact,^63^ in this study, no reduction in bacterial growth was observed for PPM-CL nanofibrous mat in the absence of NIR irradiation.

**Figure 5:**
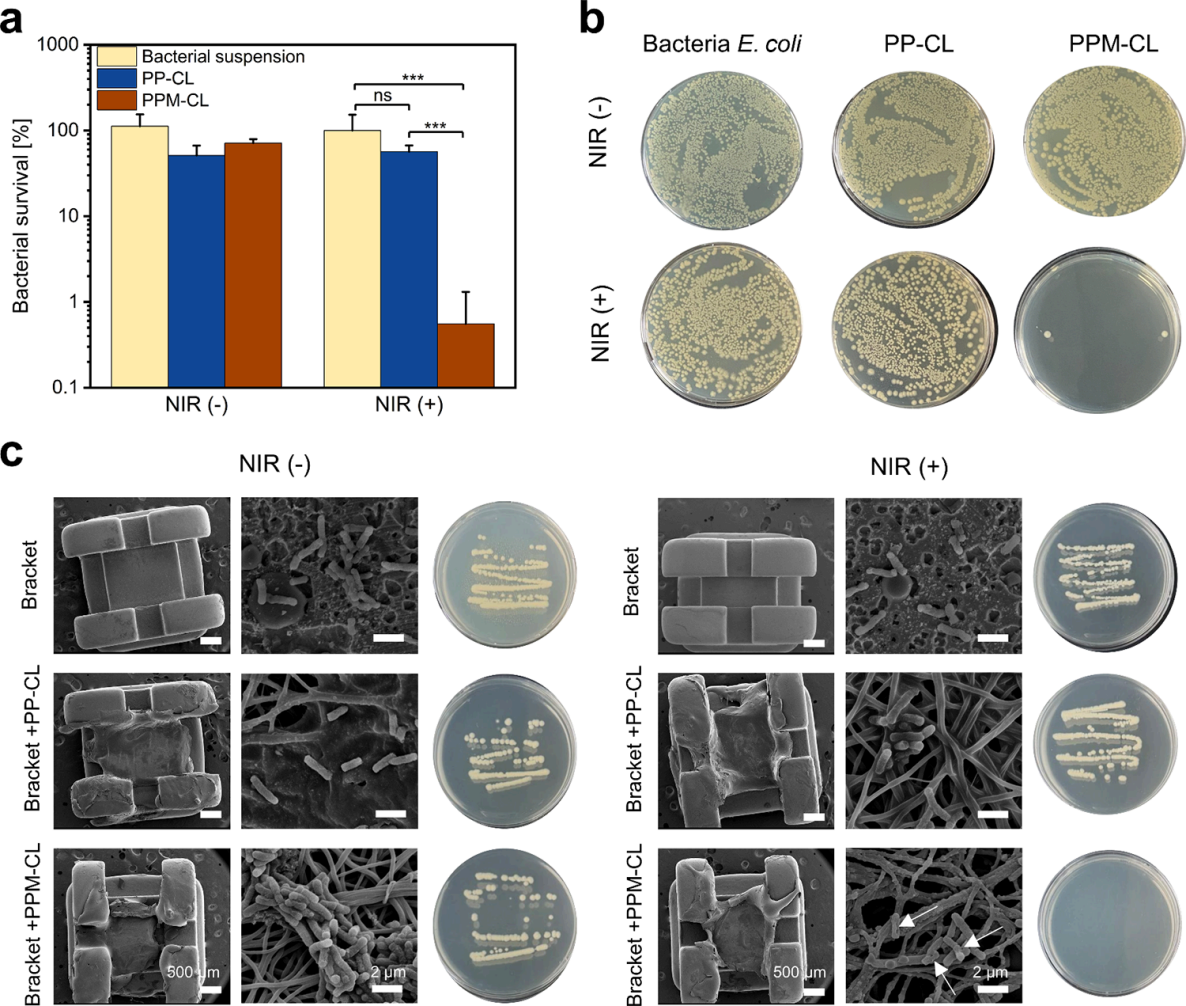
On-demand antibacterial activity of nanofibrous mats. a) Photothermal inactivation *of E. coli* on nanofibrous mats (PP-CL and PPM-CL) and in a control bacterial suspension, with and without NIR irradiation (1.5 W/cm^2^, 10 min). Results are presented on a logarithmic scale. b) Representative agar plates showing bacterial regrowth after photothermal inactivation. c) SEM images of orthodontic brackets without coating and coated with PP-CL and PPM-CL nanofibrous mats, with and without NIR irradiation. Additionally, swab samples taken from the central area of each bracket (shown on the left) were cultured on agar to assess bacterial viability.

To evaluate the performance of the system, antibacterial tests were conducted on orthodontic brackets without coatings and on brackets modified with PP-CL and PPM-CL nanofibrous mats, as shown in Figure 5c. First, the brackets were incubated with bacteria for 5 h to ensure attachment to the bracket surface. Subsequently, each bracket was either irradiated with NIR light at 1.5 W/cm^2^ for 10 min or left nonirradiated as a control.

Bacterial morphology on the bracket surfaces was examined using SEM, and swab tests were performed to assess bacterial viability. For brackets coated with PPM-CL, SEM images revealed fewer visible bacterial cells, likely due to detachment after cell death, and some remaining cells exhibited disrupted membranes, marked with white arrows (Figure 5c). Additionally, swab samples collected from the bracket surfaces coated with the PPM-CL nanofibrous mat showed no bacterial growth on agar plates. This confirms that effective bacterial eradication was achieved only through the combination of the melanin-containing nanofibrous mat with NIR irradiation.

Following the successful eradication of *E. coli*, *L. acidophilus* was evaluated. It is a probiotic species that has been frequently investigated in patients undergoing fixed orthodontic treatment.^8,64^ Recently, *Lactobacillus* research has shifted from cariogenicity toward its potential as an anticariogenic probiotic. Its role in dental caries prevention has been supported by both *in vitro* and clinical studies.^8,64^ *L. acidophilus* has also been reported to inhibit *S. mutans*, a major cariogenic bacterium commonly implicated in oral disease.^65,66^ In supplementary data (Figure S10), survival results for *L. acidophilus* show only minimal inhibition after exposure to NIR and PPM-CL. This limited effect may be explained by the relatively high thermotolerance reported for certain *L. acidophilus* strains, including survival at temperatures around 60–65 °C.^67^ The differential thermal sensitivity between *E. coli* and *L. acidophilus* underlies the selective antibacterial effect observed in our study. Selective photothermal inactivation was also demonstrated by Xu et al. Their photothermal nanoparticles selectively inactivated *methicillin-resistant Staphylococcus aureus* and *E. coli* within 5 min of treatment, while *Salmonella typhimurium* survived under the same conditions.^68^ In contrast, previous studies have shown near-complete inactivation of *S. mutans* under NIR irradiation, achieving temperatures around 55 °C for 5 min.^57,69^ Accordingly, our system may also be effective against other heat-sensitive cariogenic species.

## 3 Conclusions

In this study, electrospun nanofibrous mats incorporating MNPs derived from cuttlefish ink were developed and characterized. The resulting nanofibrous mats exhibited strong photothermal properties and functioned as an innovative antibacterial system for the surface disinfection of orthodontic brackets.

The analysis revealed that incorporating MNPs improved the electrospinning process, increased the thermal decomposition temperature by ∼77 °C, and enhanced the mechanical strength of the fibers compared with PP control, with Young’s modulus increased from 0.42 to 0.60 MPa for non-cross-linked samples. The nanofibrous mats containing MNPs exhibited an approximately 2-fold increased swelling capacity and a reduced contact angle, indicating improved hydrophilicity. Good antioxidant activity was also observed for the PPM-CL nanofibrous mat, reaching approximately 60% DPPH radical scavenging after 10 h. Under NIR irradiation at 1.5 W/cm^2^, the nanofibrous mats reached ∼101 °C in dry conditions and approximately 55–60 °C in wet conditions, temperatures sufficient for photothermal bacterial deactivation. The nanofibrous mats containing MNPs (PPM-CL) were suitable for biomedical applications, as testing on L929 fibroblast cells showed no cytotoxicity and supported normal cell growth.

Most importantly, our study demonstrated that PPM-CL nanofibrous mats containing MNPs, when irradiated with an NIR laser, effectively eradicated bacteria, specifically targeting *E. coli* as a model organism, reducing survival to 0.55%. It was also confirmed that nanofibrous mats placed on the inner surface of orthodontic brackets effectively damaged bacterial membranes under irradiation. Additionally, testing against *L. acidophilus*, a bacterium commonly associated with orthodontic appliances, indicated only minor inhibitory effects.

In conclusion, the findings underscore the potential of these electrospun nanofibrous mats containing MNPs for practical applications in antimicrobial treatments and other biomedical fields, providing a flexible, efficient, and nontoxic solution for PTT and surface disinfection. Notably, the use of melanin derived from cuttlefish ink embedded in nanofibrous mats for the photothermal eradication of bacteria represents a novel approach, as previous studies have predominantly employed nanofibrous mats for other applications. This innovation highlights the unique properties and advantages of cuttlefish ink for the development of effective antibacterial systems and underscores the need for new solutions to disinfect orthodontic brackets.

Future work could also explore alternative MNP morphologies, such as ellipsoidal, hollow, or mesoporous melanin nanoparticles, which may further enhance photothermal conversion efficiency through increased surface-area-to-volume ratios and could additionally serve as drug-loading reservoirs for combined photothermal-chemotherapeutic strategies.^15^

While this system has shown potential to improve hygiene, it is important to investigate whether the nanofibrous mats, after irradiation, pose any risks from residual bacteria. Additionally, the influence of everyday hygiene practices, such as brushing, on fiber stability and retention should be examined. Further, *in vivo* studies involving a broader spectrum of oral bacteria are also necessary. Beyond orthodontic brackets, this system may be applicable in other biomedical and antimicrobial applications.

## 4 Materials and Methods

### 4.1 Materials

Cuttlefish ink was purchased as sachets from Nortindal (Spain). Chemicals and reagents used in this study were sourced from several suppliers. Poly­(vinyl alcohol) (PVA) with an average molecular weight of 85 000–124 000 Da and a degree of hydrolysis of 99+%, poly­(ethylene oxide) (PEO) with an average molecular weight of 1 000 000 Da, 2,2-diphenyl-1-picrylhydrazyl (DPPH), hexamethyldisilazane (HMDS), glutaraldehyde (grade I), paraformaldehyde (reagent grade) and ethanol absolute, were all obtained from Sigma-Aldrich. Methanol pure P.A. was purchased from POCH. Adhesive glue (Poxipol, components A and B). Phosphate buffered saline (PBS), triton X-100, and 4′,6-diamidino-2-phenylindole dihydrochloride (DAPI) were purchased from Roth. Dulbecco’s modified eagle’s medium (DMEM), fetal bovine serum (FBS), penicillin-streptomycin (P/S), and EDTA-Trypsin were provided by Gibco Invitrogen. Alexa Fluor 488 Phalloidin, Live/Dead Viability/Cytotoxicity Kit, and PrestoBlue reagent were supplied by Thermo Fisher Scientific. Lysogeny broth (LB) and lysogeny agar (LB Agar) were acquired from A&A Biotechnology. De Man, Rogosa and Sharpe (MRS) agar and broth were purchased from Formedium. *Escherichia coli* (ATCC 25922) and *Lactobacillus acidophilus (ATCC 4356)* were bought from Pol-AUR. Standard edgewise orthodontic brackets were purchased from HRRSDENTAL Store.

### 4.2 Methods

#### 4.2.1 Extraction of Melanin from Cuttlefish Ink

Cuttlefish ink (4 g) was placed in a Falcon tube, and 45 mL of deionized (DI) water was added. The samples were vortexed for 5 min. The Falcon tubes were then centrifuged at 2000 rpm for 15 min, after which the supernatant was replaced with fresh DI water. This procedure was repeated five times to ensure the purification of melanin nanoparticles from salts. Subsequently, the suspension was freeze-dried at −80 °C for 2 days. To prepare the solution for electrospinning, the freeze-dried melanin was crushed using a mortar. The final suspension was prepared at a concentration of 25 mg/mL in a 50:50 water:ethanol mixture.

#### 4.2.2 Fabrication of Electrospun PVA/PEO Nanofibrous Mat with MNPs

Based on our previous optimization study,^19^ a PVA/PEO blend was selected as the electrospinning matrix. PEO was incorporated to enhance the viscoelastic properties of the spinning solution and improve jet stability during electrospinning, particularly after addition of the melanin nanoparticle suspension, which reduced the effective polymer concentration due to dilution. The base solution of PVA/PEO was prepared by dissolving these polymers in a ratio of 7:3 in Milli-Q deionized water to achieve a 10% w/v concentration. This mixture was heated and stirred at 90 °C for 3 h to ensure complete dissolution. Subsequently, the solution was allowed to mix overnight to attain homogeneity. The final electrospinning solutions (PP and PPM) were prepared by mixing the base PVA/PEO solution with either DI water/ethanol or the melanin nanoparticle suspension, as detailed in Table 1. The optimization of the melanin suspension addition to the polymer solution and electrospinning parameters is described and presented in Table S1, Figures S11–S13 of the Supporting Information.

**Table 1:** Mixing Ratios of the Electrospinning Solutions

Sample	10% PVA/PEO (parts by vol.)	DI water/Ethanol (50:50) (parts by vol.)	MNPs (25 mg/mL) (parts by vol.)
PP	1	0.75	
PPM	1		0.75

For the electrospinning procedure, each PP and PPM solution was loaded into a 1 mL syringe fitted with a 22G needle (internal diameter of 0.413 mm). The syringe was mounted on a syringe pump, dispensing the polymer solution at a flow rate of 200 μL/h. A high-voltage power supply set to 18 kV facilitated the formation of nanofibers, which were collected on a rotating collector positioned 16 cm from the needle tip at a rotation speed of 400 rpm. Electrospinning was conducted under controlled ambient conditions, with a room temperature and a relative humidity of 45%.

#### 4.2.3 Morphological Characterization

Cuttlefish ink and nanofibrous mats (PP, PPM, PP-CL and PPM-CL) were imaged using a Field Emission Scanning Electron Microscopy (FE-SEM) (ZEISS Crossbeam 350 FIB-SEM microscope). Before imaging, nanofibrous mats were sputtered with gold (6 min) in a DII-29030SCTR JEOL Smart Coater.

To confirm melanin incorporation into the nanofibers, an electrospinning solution containing 25 mg/mL of previously prepared melanin nanoparticle suspension was electrospun directly onto TEM grids for scanning transmission electron microscopy (STEM) analysis (ZEISS Crossbeam 350 microscope). Nanofibrous mats were collected on the grids during electrospinning. For cross-linking, the nanofibrous mats were placed in an oven at 150 °C for 2 h prior to visualization.

Nanofibrous mats in Supporting Information (Figures S11–S13), L929 cells from the biocompatibility assay, and bacteria on orthodontic brackets were imaged using a scanning electron microscope (SEM) (JSM-6010PLUS/LV, InTouchScope). Prior to imaging, samples were sputter-coated with gold for 6 min.

#### 4.2.4 Dynamic Light Scattering (DLS) Analysis and Zeta Potential

The size distribution of MNPs was determined using the DLS method with a Zetasizer Nano ZS (model ZEN3600, Malvern Instruments, Malvern, UK). The analysis was conducted on a 1 mg/mL suspension in DI water. Each sample was measured in triplicate following sonication and a 1 min stabilization period at 25 °C.

#### 4.2.5 Attenuated Total Reflectance Fourier-Transform Infrared (ATR-FTIR) Spectroscopy

ATR-FTIR spectroscopy was employed to investigate the chemical bonds and verify the chemical structure of the composite material and its constituent components. Spectra were recorded in the 400–4000 cm^–1^ range with a resolution of 2 cm^–1^ using VERTEX 70 (Bruker) equipment. Spectra were collected for PP and PPM before and after cross-linking, as well as for cuttlefish ink powder, to confirm the successful incorporation of melanin.

#### 4.2.6 Thermal Analysis

TGA was conducted on a TA Instruments Q600 apparatus operating in air flux at a heating rate of 10 °C/min; a TA Instruments Q2000 differential scanning calorimeter was used for DSC characterizations under a nitrogen atmosphere at a heating scan of 10 °C/min.

#### 4.2.7 Mechanical Properties

To investigate the influence of MNPs on the mechanical properties of PVA/PEO nanofibrous mats, a comprehensive tensile characterization was performed for PVA/PEO mats without MNPs (PP) and with MNPs (PPM), both before and after cross-linking. Measurements were carried out using a CTX texture analyzer (AMETEK Brookfield, USA) equipped with a 10 N load cell and grips adapted for thin and delicate specimens. Rectangular strips of electrospun nanofibrous mats (10 × 40 mm) were carefully mounted to ensure accurate alignment and uniform stress distribution during testing. The thickness of each nonwoven sample was measured using Kroeplin flat calipers (estimated measurement error: 0.003 mm), and the average thickness value was used for stress calculations. Tensile tests were conducted at a constant crosshead speed of 0.5 mm/s. Stress–strain curves were analyzed to determine Young’s modulus, tensile strength, and elongation at break. Each formulation was tested in triplicate, and all measurements were performed on dry samples under ambient conditions.

#### 4.2.8 Swelling with Degradation and Melanin Release Studies

Approximately 10 mg of each sample, including material and material/melanin, were accurately weighed and transferred into 2 mL Eppendorf tubes. Each sample was immersed in 1 mL of PBS (pH 7.4) and incubated at 37 °C. At predetermined time points, the samples were removed, thoroughly dried using tissue, and reweighed. The supernatant was measured by UV spectrophotometry for melanin release and then replaced with a fresh portion of PBS. The samples were measured at 270 nm wavelength (melanin absorption peak) (Figure S14a). The material samples without melanin, at the same weight, were used as a reference to avoid polymer interference. The melanin concentrations were determined using a calibration curve presented in Figure S14b (*R* ^2^ = 0.997) established after confirming the maximum absorption peak of melanin in standard melanin/PBS solutions of known concentrations.

#### 4.2.9 Water Contact Angle Measurement

Surface wettability was assessed by measuring contact angles using a Data Physics OCA 15EC instrument (Germany). Three independent water contact angle measurements were performed on each sample. The samples were placed on a microscope glass slide, and a 5 μL water droplet was deposited on them at room temperature. Images of the droplets on the material were captured 0.3 s after application. The contact angle was then measured using ImageJ with LBADSA plugin. Photographs of the glass slide were taken, and the droplet spread diameter was measured using ImageJ.

#### 4.2.10 Antioxidant Activity

The free radical-scavenging ability of melanin was evaluated using the DPPH assay. The material containing melanin, the reference material, and blank samples (DPPH solution) were placed in a 96-well plate. A 23.6 mg/L DPPH was prepared in methanol, and 100 μL was dispensed into each well containing the tested material, as well as into control wells. The plate was incubated at 37 °C for 2 h, 4 h, 8 h, 24 h, and 48 h. After each time point, the medium was collected, and the sample absorbance was recorded at λ = 517 (corresponding to the DPPH maximum absorbance wavelength). The ROS scavenging ability was calculated as the reduction in the absolute absorbance value. All measurements were performed in triplicate, and mean values were calculated.

#### 4.2.11 Photothermal Properties

To evaluate the photothermal properties of melanin incorporated into the nanofibrous mats, samples were exposed to NIR laser irradiation at a wavelength of 808 nm. Circular nanofibrous mats with a diameter of 1.1 cm were prepared using a puncher and placed individually into a 48-well plate. Photothermal measurements were performed under both dry conditions and wet conditions. For wet conditions, 100 μL of DI water was added to the nanofibrous mats.

The samples were irradiated at power densities of 1.0, 1.5, and 2.0 W/cm^2^. Temperature changes were recorded in real time using a high-resolution infrared thermal camera (FLIR A655sc, FLIR Systems), and thermal data were analyzed with FLIR ResearchIR Max software. The maximum surface temperature (*T*_max_) and temperature–time profiles were extracted from the region of interest corresponding to the nanofiber-covered area. To assess photothermal stability and repeatability, PPM-CL was subjected to 4 irradiation cycles. Each cycle consisted of 2 min of laser exposure followed by 2 min of passive cooling at room temperature.

For evaluation in a clinically relevant configuration, an extracted human tooth was used as a substrate. An orthodontic bracket was bonded to the enamel surface using a two-component adhesive glue. The lower half of the tooth was partially immersed in water to simulate intraoral wet conditions. Nanofibrous samples were attached to the bracket surface, and temperature measurements were recorded specifically from the nanofiber-covered region during NIR irradiation.

#### 4.2.12 *In Vitro* Biocompatibility Study

L929 murine fibroblast cells were grown in DMEM supplemented with 10% FBS and 1% P/S, and maintained in an incubator set to 37 °C with 5% CO_2_. The culture medium was replaced every 2 days. Cells were passaged when they reached approximately 80% confluence. For seeding, cells were detached using 0.05% EDTA-trypsin for 3 min, followed by incubation at 37 °C with 5% CO_2_. The cells were then collected in a Falcon tube and centrifuged at 1200 rpm for 5 min, forming a pellet at the bottom of the tube. The cells were resuspended in 1 mL of culture medium and counted.

Nanofibers were electrospun onto circular glass slides (diameter of 1.5 cm) and subsequently cross-linked at 150 °C for 2 h. The cross-linked samples (PP-CL and PPM-CL) and clean glass slides (control) were sterilized under UV light for 30 min per side and placed into a 24-well plate. Cells were then seeded directly onto the substrates at a density of 10,000 cells per well in 1 mL of culture medium. The medium was replaced on day 3, and the cultures were maintained for a total of 7 days.

##### 4.2.12.1 Cell Proliferation

Cell viability was quantified using the PrestoBlue proliferation assay on days 1, 3, and 7 of culture. Samples and controls (*n* = 5 per group) were incubated with 10% (v/v) PrestoBlue reagent diluted in culture medium for 2 h at 37 °C and 5% CO_2_. Subsequently, three 100 μL aliquots from each well were transferred to a 96-well plate, and fluorescence was measured at an excitation wavelength of 530 nm and an emission wavelength of 620 nm using a microplate fluorometer (Fluoroskan Ascent Microplate Fluorometer, Thermo Scientific, USA).

##### 4.2.12.2 Scanning Electron Microscopy Morphological Visualization

Prior to SEM imaging, the culture medium was removed and the samples were washed with PBS. Samples were fixed in ice-cold 3% glutaraldehyde for 20 min, washed three times with deionized water, and dehydrated by sequential immersion in ethanol solutions of increasing concentration (25%, 50%, 75%, and 100%) for 15 min each. The samples were then treated with a graded series of HMDS in ethanol (25%, 50%, 75%, and 100%) for 15 min each. Finally, samples were air-dried overnight under a fume hood. SEM analysis was performed in triplicate for each condition at day 3 and day 7 after seeding.

##### 4.2.12.3 Live/Dead Staining

First, the cells were washed with PBS and incubated for 10 min in a dye solution containing 0.5 μL of calcein (staining live cells green) and 2 μL of ethidium homodimer (staining dead cells red) in 1 mL of sterile PBS. After washing the cell samples three times with PBS, confocal microscopy (TCS SP5 X, Leica, Germany) was performed. This test was performed in triplicate for each condition at day 3 and day 7 after seeding.

##### 4.2.12.4 Cytoskeleton and Nuclei Visualization

For Actin/Dapi staining, samples were washed with PBS and fixed in 4% paraformaldehyde for 30 min at room temperature. Then samples were washed three times with PBS and treated with a 0.3% (v/v) Triton X-100 solution for 15 min. Following another wash, the samples were incubated in a solution of 1% (w/v) BSA with 0.1% Triton X-100 for 1 h. Next, the samples were washed and incubated with a mixture of 1% BSA, 0.1% Triton X-100, and a 1:40 solution of Alexa Fluor 488 Phalloidin for 1 h in the dark. Finally, the nuclei were stained with a 1:500 dilution of DAPI in PBS for 10 min. The samples were washed three times, and the cell cytoskeleton and nuclei were visualized using a confocal microscope (TCS SP5 X, Leica, Germany). This test was performed in triplicate for each condition at day 3 and day 7 after seeding.

#### 4.2.13 Bacteria Study

Colonies of *Escherichia coli* (ATCC 25922) were grown in LB. Colonies of *Lactobacillus acidophilus (ATCC 4356)* were cultured in MRS broth. For experiments, overnight cultures were grown at 37 °C in the corresponding broth medium until reaching an optical density at 600 nm (OD_600_) of ∼1.0. Bacterial suspensions used for all studies were prepared by diluting OD_600_ of ∼1.0 stock culture in sterile PBS to a final concentration of 1 × 10^6^ CFU/mL.

##### 4.2.13.1 Photothermal Test of Nanofibrous Mats with Bacteria

Before antibacterial testing, nanofibrous mats (PP-CL and PPM-CL) were sterilized under UV light for 30 min per side. Three replicates of each sample for each condition were placed in a 48-well plate for NIR irradiation experiments. Each well received 100 μL of bacterial suspension (either *E. coli* or *L. acidophilus*) and was used for subsequent irradiation or control conditions. Control wells without nanofibrous mats received 100 μL of bacterial suspension only.

NIR irradiation parameters were first optimized using *E. coli* by testing irradiation times of 5, 7, 10, and 15 min. Control samples (nonirradiated) were incubated at room temperature for the same period. For *L. acidophilus*, only a 10 min irradiation time was evaluated. Immediately after irradiation, 100 μL of sterile PBS was added to each well, and the bacterial suspension was resuspended by pipetting. Suspensions were transferred to a 96-well plate and serially diluted in PBS. For CFU quantification, 5 μL aliquots of each dilution were plated in triplicate onto LB agar (for *E. coli*) or MRS agar (for *L. acidophilus*). Plates were incubated under appropriate conditions until colonies were visible, and bacterial survival was determined by colony counting.

For macroscopic visualization in the *E. coli* study, the bacterial suspension recovered from the 48-well plate was diluted 10-fold, evenly spread onto LB agar plates, incubated, and photographed for qualitative comparison.

##### 4.2.13.2 Photothermal Evaluation of Nanofibrous Mat-Coated Orthodontic Brackets Using *E. coli*

Sterilized nanofibrous mats were cut to the desired shape and attached to brackets using a minimal amount of adhesive glue. The assembled brackets with nanofibrous mats were sterilized again under UV light for 30 min.

Brackets were placed into a 96-well plate, and 100 μL of the prepared *E. coli* suspension (1 × 10^8^ CFU/mL) was added to each well. Samples were incubated for 5 h. After incubation, 75 μL of the suspension was removed to maintain a moist environment, and each well was irradiated for 10 min using NIR light at 1.5 W/cm^2^.

After irradiation, the inner surface of each bracket (where the nanofibrous mats were located) was sampled by touching four distinct locations with a sterile spatula. Then the spatula was streaked onto LB agar. Plates were incubated to assess bacterial growth, and the plates were photographed for qualitative comparison. This test was done for two replicates.

The brackets prepared for SEM were processed after removing the residual bacterial suspension. Ice-cold 3% glutaraldehyde was added to fully cover each bracket, and the samples were fixed for 12 h at 4 °C. Later, the brackets were washed three times with DI water and dehydrated using a graded ethanol series (10%, 25%, 50%, 75%, 90%, and 100%). After dehydration, samples were dried prior to SEM imaging. Before imaging, brackets with bacteria were sputtered with gold.

#### 4.2.14 Statistical Analysis

All data were presented as mean ± standard deviation (SD). A one-way analysis of variance (ANOVA) with (Tukey’s test) was used to determine differences (*p*-value ≤ 0.05: *p ≤ 0.05, **p ≤ 0.01, ***p ≤ 0.001), test calculated by OriginPro software.

## Supplementary Material



## Notes

Photography of purified melanin nanoparticles (MNPs) isolated from cuttlefish ink, ATR-FTIR spectrum of MNPs confirming their characteristic functional groups, STEM image of a cross-linked PVA/PEO nanofibrous mat (PP-CL), ATR-FTIR spectra of as-spun nanofibrous mats, mass change of nanofibrous mats after immersion in PBS, release profile of melanin nanoparticles from nanofibrous mats in water at different time points, SEM images of cross-linked nanofibrous mats after incubation in water collected over several days, antibacterial activity studies showing the survival of *Escherichia coli* after NIR irradiation at different exposure times, survival of *Lactobacillus acidophilus* after 10 min of NIR irradiation (1.5 W/cm^2^), photographs of as-spun electrospun samples prepared with different MNP loadings and SEM images of electrospun nanofibrous mats prepared with and without melanin nanoparticles, optimization of electrospinning parameters for the control PVA/PEO formulation without MNPs, UV–vis absorption spectrum of melanin nanoparticles in PBS and corresponding calibration curve, and detailed composition of electrospinning solutions with varying MNP suspension contents (PDF)
