# Supplementary material for: Cuttlefish Ink-Derived Melanin Nanoparticles Enabling NIR-Responsive Electrospun Nanofibrous Mats for On-Demand Selective Antibacterial Disinfection of Orthodontic Braces
Source: ACS Appl Mater Interfaces. 2026 Jun 1;18(22):30940–55. doi: 10.1021/acsami.6c05032 (PMC13308878; doi:10.1021/acsami.6c05032)
Supplement: Supplementary file 1 [file am6c05032_si_001.pdf]

Supporting Information

Cuttlefish Ink-Derived Melanin Nanoparticles Enabling NIR-Responsive Electrospun Nanofibrous Mats for On-Demand Selective Antibacterial Disinfection of Orthodontic Braces

*Magdalena Bartolewska[1], Daniel Rybak[1], Alicja Kosik-Kozioł[1], Piotr Jenczyk[2], Michał Pruchniewski[3], Dariusz Jarząbek[2], Massimiliano Lanzi[4], and Filippo Pierini[1]**

[1] Department of Biosystems and Soft Matter, Institute of Fundamental Technological Research, Polish Academy of Sciences, Warsaw, 02–106 Poland

[2] Department of Mechanics of Materials, Institute of Fundamental Technological Research, Polish Academy of Sciences, Warsaw 02-106, Poland

[3] Department of Nanobiotechnology, Institute of Biology, Warsaw University of Life Sciences, Warsaw 02-786, Poland.

[4] Department of Industrial Chemistry "Toso Montanari", University of Bologna, Viale Risorgimento 4, Bologna, 40136 Italy

* Corresponding author's e-mail: fpierini@ippt.pan.pl

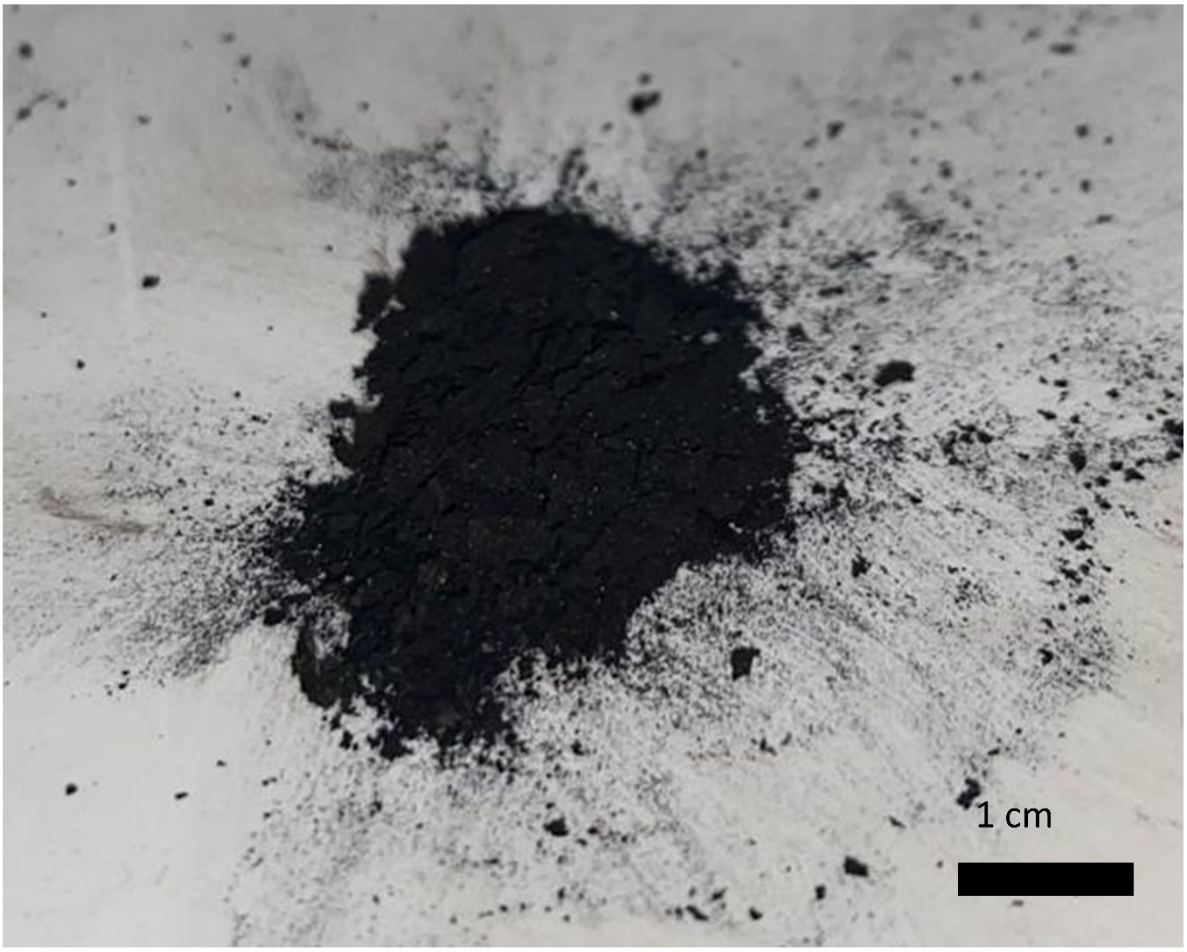

Figure S1. Image of purified melanin nanoparticles (MNPs) from cuttlefish ink.

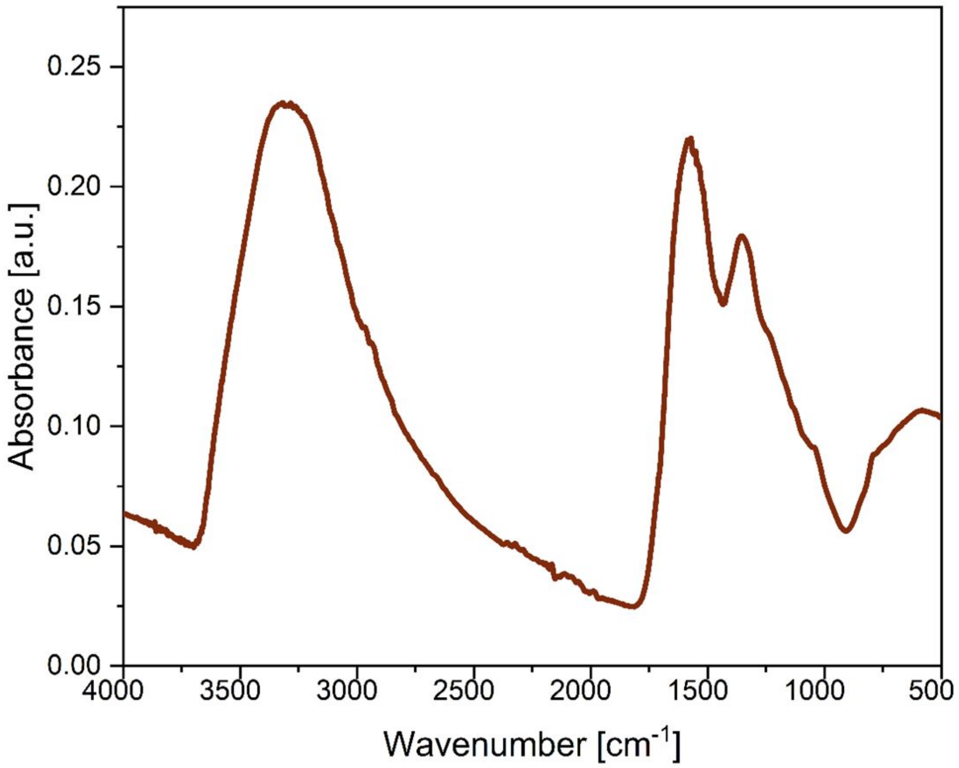

Figure S2. FTIR-ATR of MNPs.

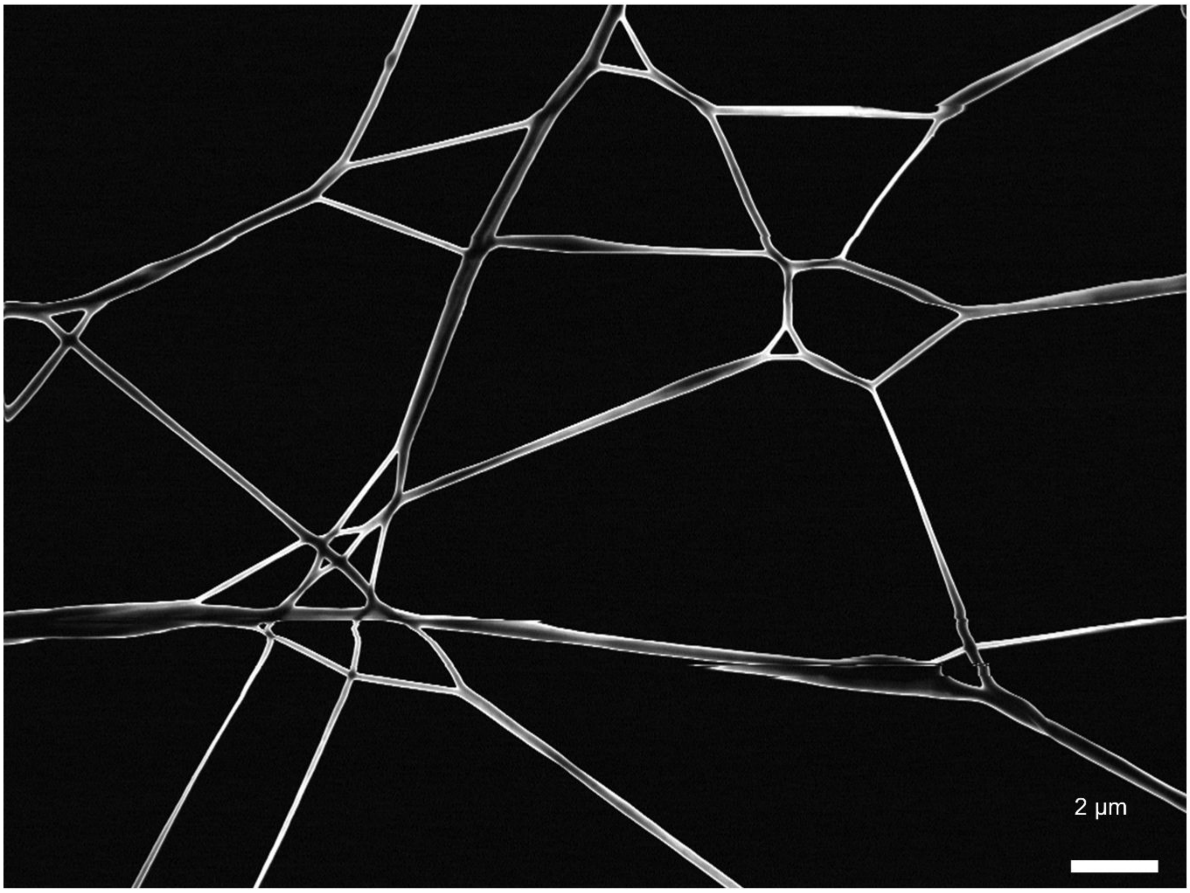

Figure S3. STEM image of PVA/PEO crosslinked nanofibrous mat (PP-CL).

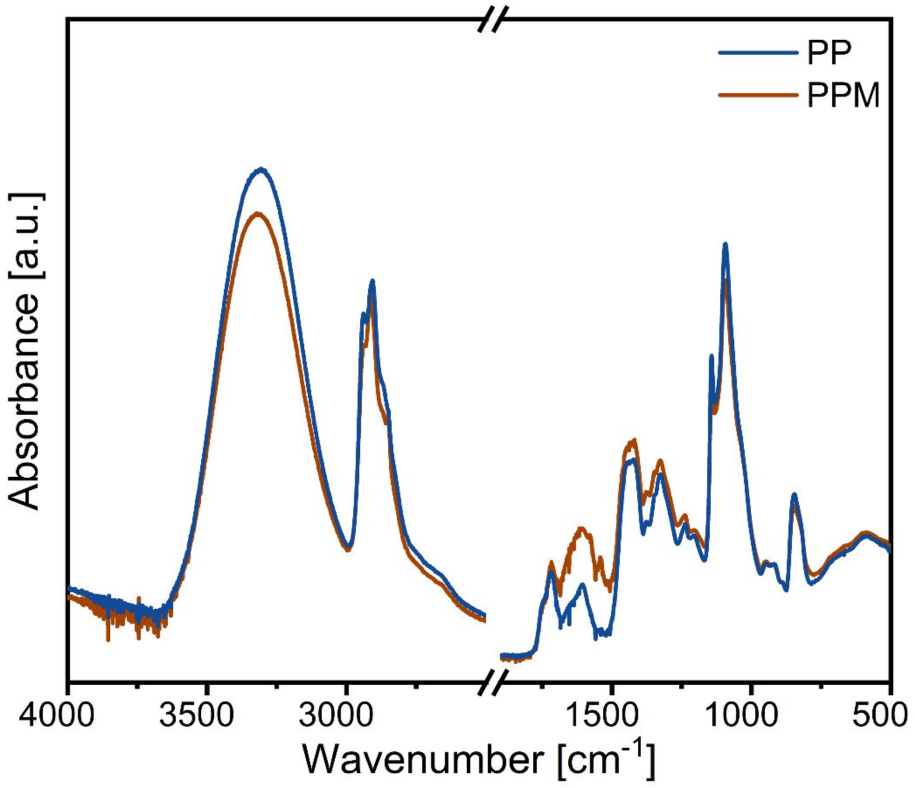

Figure S4. ATR-FTIR of as-spun nanofibrous mats.

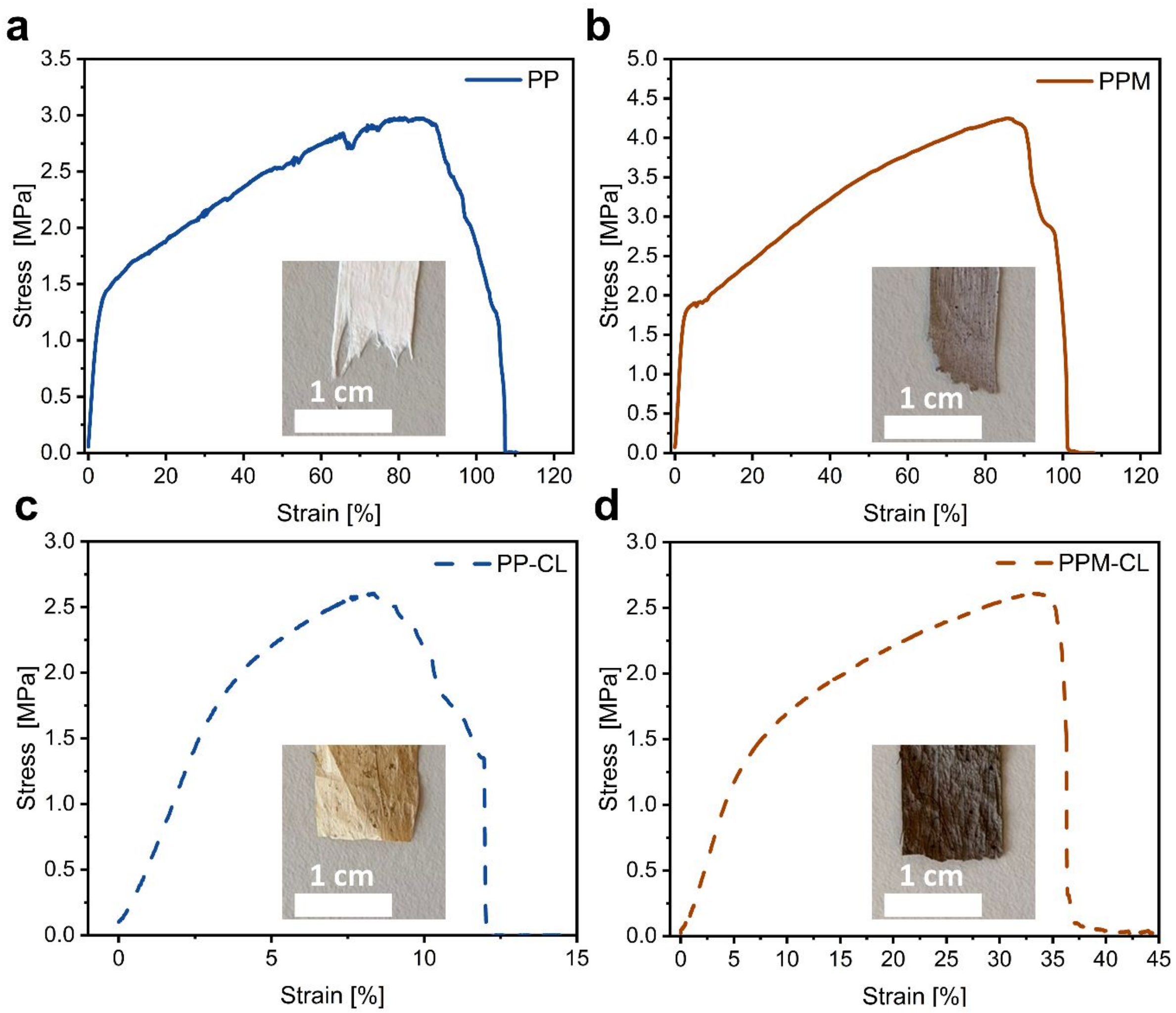

Figure S5. Stress–strain curves illustrating the mechanical behavior of the nanofibrous mats, with inset photographs showing the fracture ends after tensile testing a) PP, b) PPM, c) PP-CL, and d) PPM-CL.

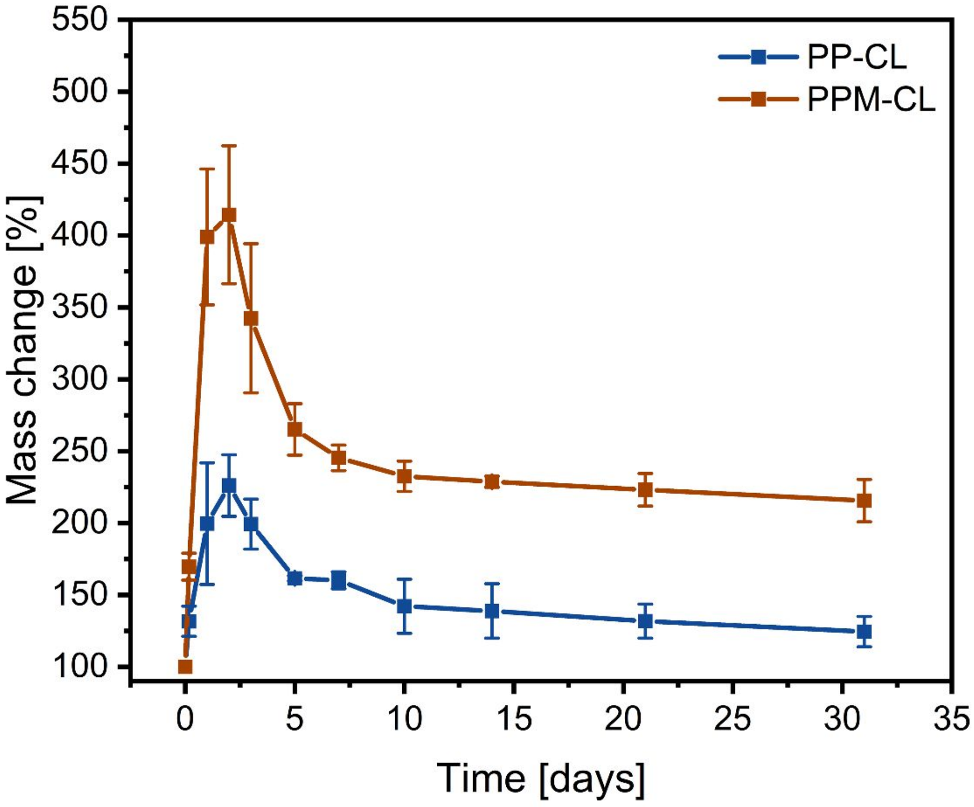

Figure S6. Mass change of nanofibrous mats after immersion in PBS.

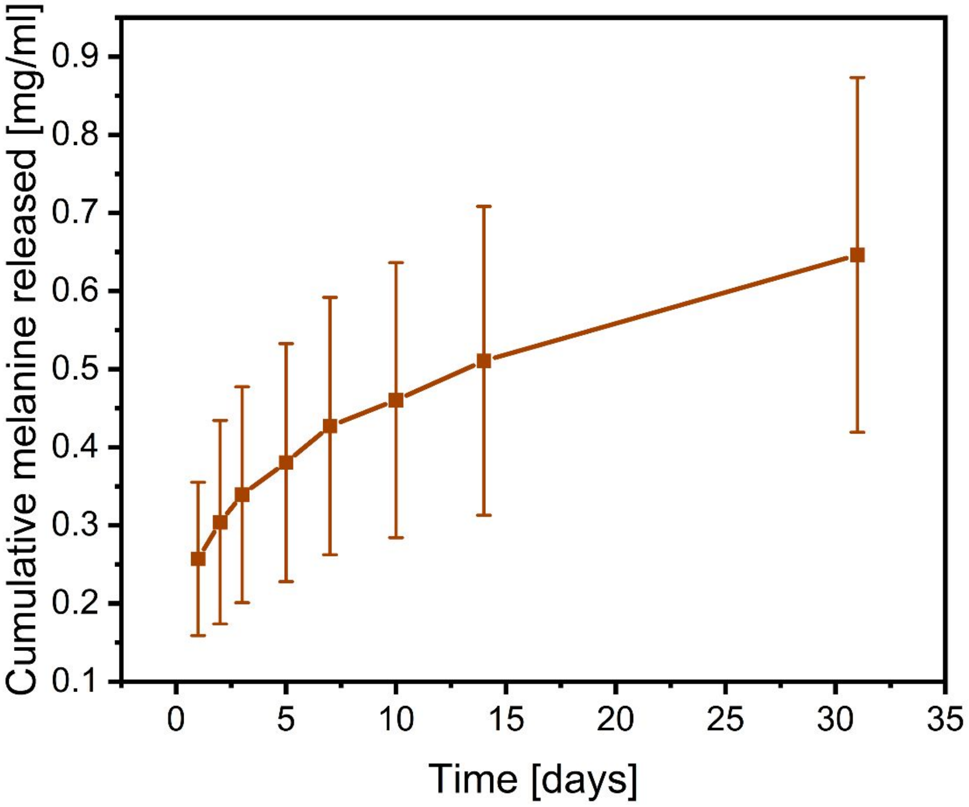

Figure S7. Release of melanin nanoparticles after immersion of nanofibrous mats in PBS at different time points.

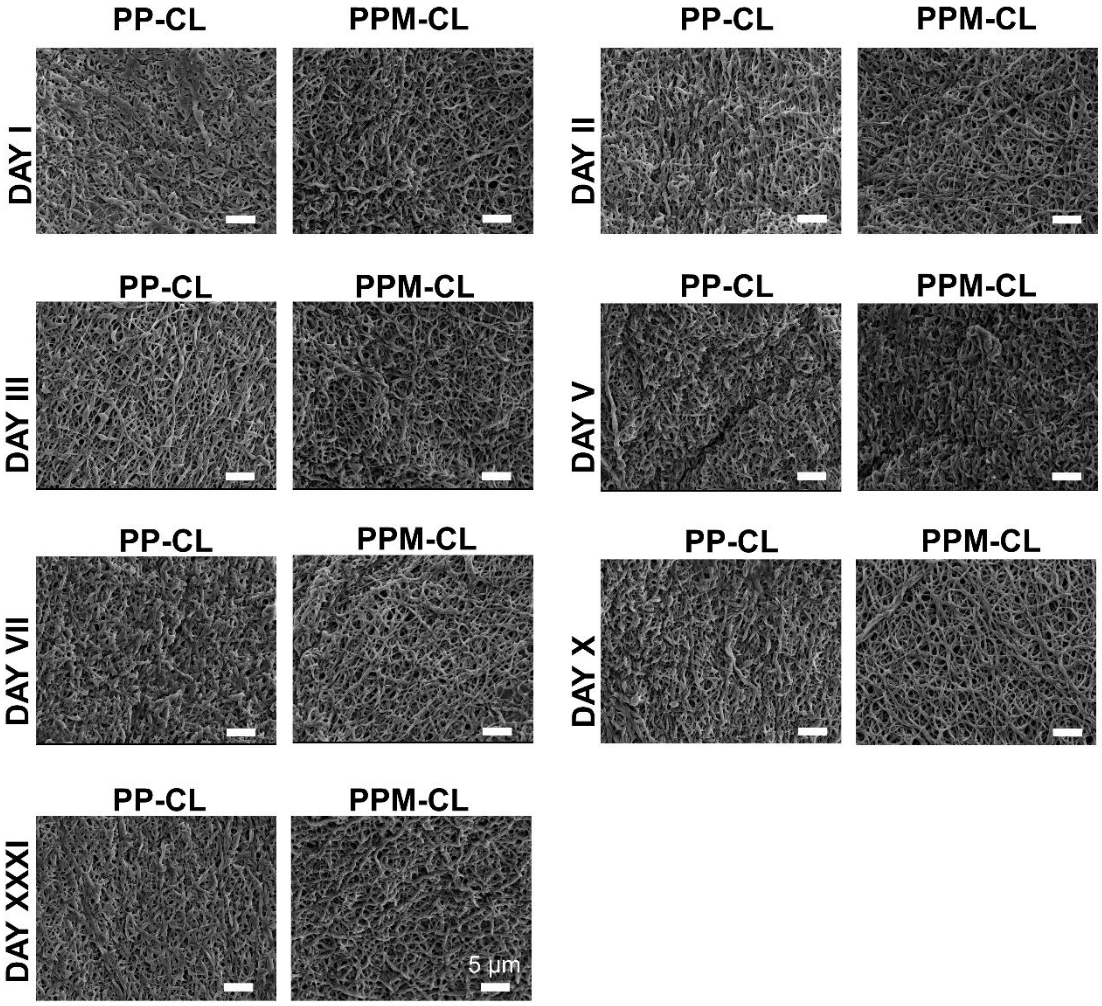

Figure S8. SEM images of crosslinked nanofibrous mats after incubation in PBS, collected on different days.

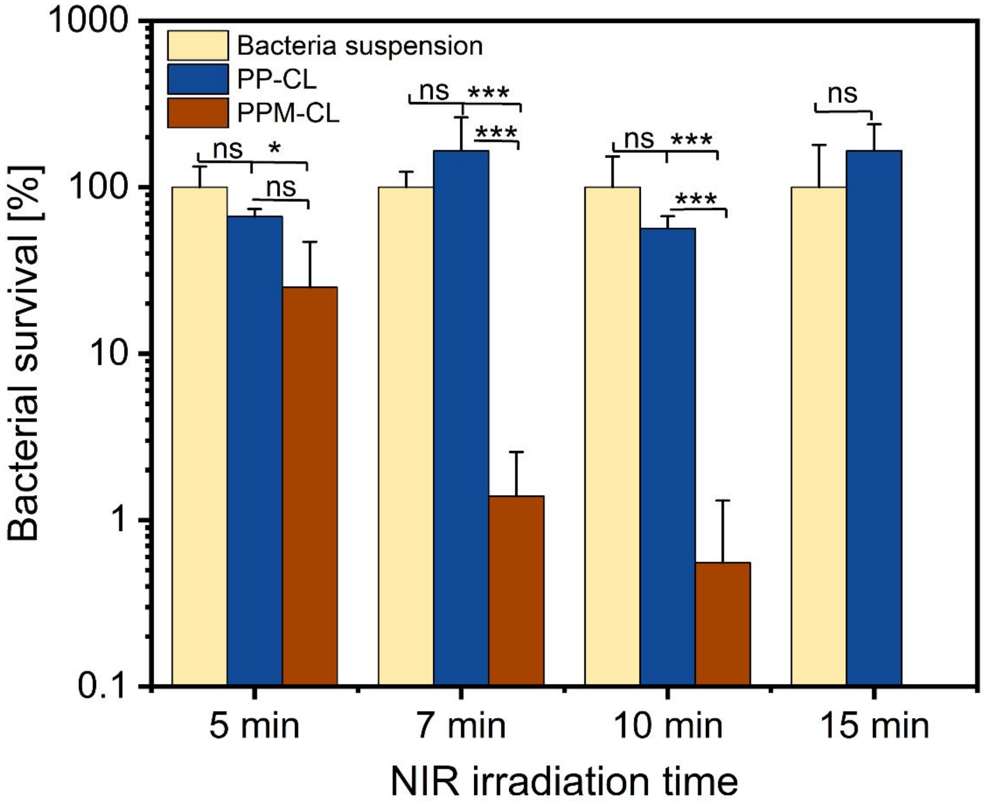

Figure S9. Survival of *E. coli* after NIR irradiation at different exposure times.

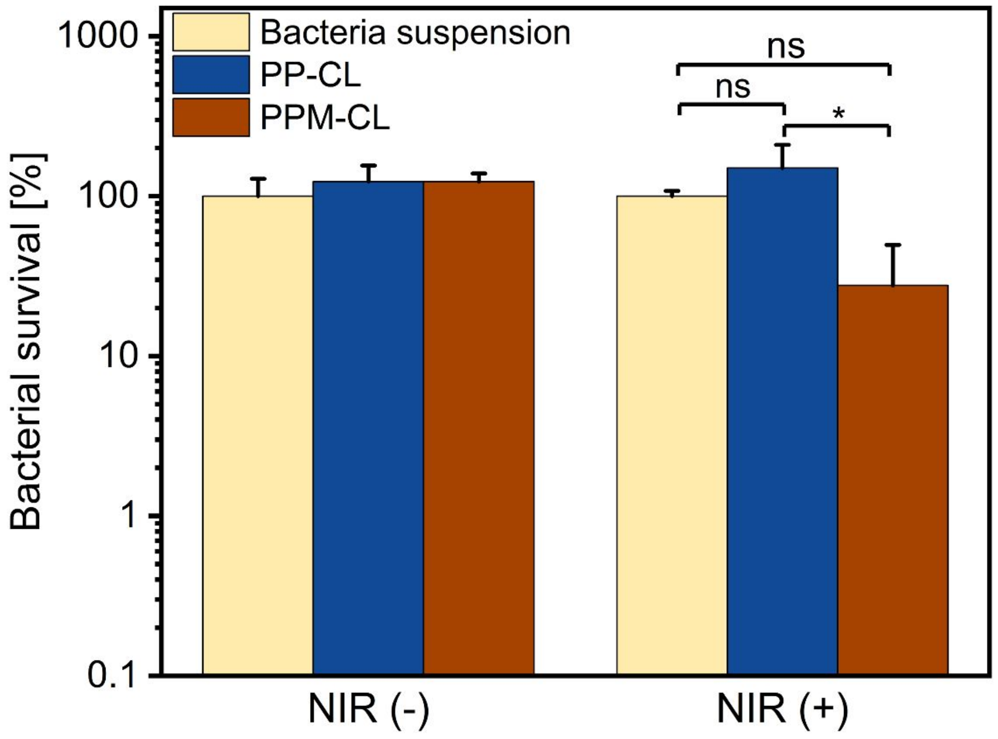

Figure S10. Survival of *L. acidophilus* after 10 min NIR irradiation (1.5 W/cm^2).

For the optimization of electrospun nanofibrous mats, a 10% PVA/PEO solution prepared in DI water was used. Various amounts of a melanin nanoparticle (MNPs) suspension (25 mg/mL in ethanol/H_2O) were added. Different volumes of the suspension were mixed with 1 mL of polymer solution. This was done to determine the maximum dilution of PVA/PEO suitable for electrospinning and the highest MNPs loading achievable (Table S1).

A 50 mg/mL suspension was also tested but is not reported here. This suspension rapidly precipitated and formed sediment at the bottom of the vial, indicating poor stability. Before addition to each PVA/PEO solution, the MNPs suspension was sonicated for 30 min and vortexed for several minutes to improve dispersion.

All solutions listed in Table S1 were electrospun at 16 kV using a 22G needle, a tip-to-collector distance of 16 cm, and a flow rate of 400 μL/h. Photographs and SEM images of the

nanofibers are shown in Figure S11. Even Samples 5 and 6, which contained a 50% dilution of PVA/PEO, produced smooth fibers with visible small melanin nanoparticles.

The same parameters did not produce satisfactory fibers for the corresponding control samples (Samples 5 and 6). Instead of MNPs, an equal volume of H_2O/EtOH (50:50) was added. The resulting fibers are presented in Figure S12. Under identical conditions, the control solutions produced beaded fibers. Sample 6 formed a thin mat with numerous beads and droplets.

Further optimization was performed using the composition of Sample 5 (1 mL PVA/PEO and 0.75 mL MNPs suspension). To improve the control sample without melanin, additional electrospinning parameters were evaluated.

As shown in Figure S13a, the voltage (16 kV) and flow rate (400 µL/h) were first maintained. Reducing the flow rate to 200 µL/h significantly improved fiber formation and reduced bead defects (Figure S13b). The most uniform bead-free fibers were obtained at 18 kV and 200 µL/h. These conditions were selected as the optimized parameters.

The results suggest that MNPs stabilize the electrospinning process. Bead-free nanofibers were obtained even at 400 µL/h in the presence of MNPs. For consistency and comparison across all experiments in the main manuscript, all fibers were prepared using 18 kV and 200 µL/h.

Table S1. Composition of electrospinning solutions used for sample preparation, with a constant polymer solution volume (PVA/PEO, 10% w/v) and varying volumes of MNPs suspension (25 mg/mL in 50:50 DI water:ethanol).

**Sample nr.**	**PVA/PEO (10%w/v)**	**MNPs suspension (25 mg/mL, 50:50 DI water:ethanol)** *For the control solution, the MNPs suspension was replaced with an equal volume of 50:50 DI water:ethanol.
1	1 mL	-
2	1 mL	0,1 mL
3	1 mL	0,2 mL
4	1 mL	0,5 mL
5	1 mL	0,75 mL
6	1 mL	1 mL

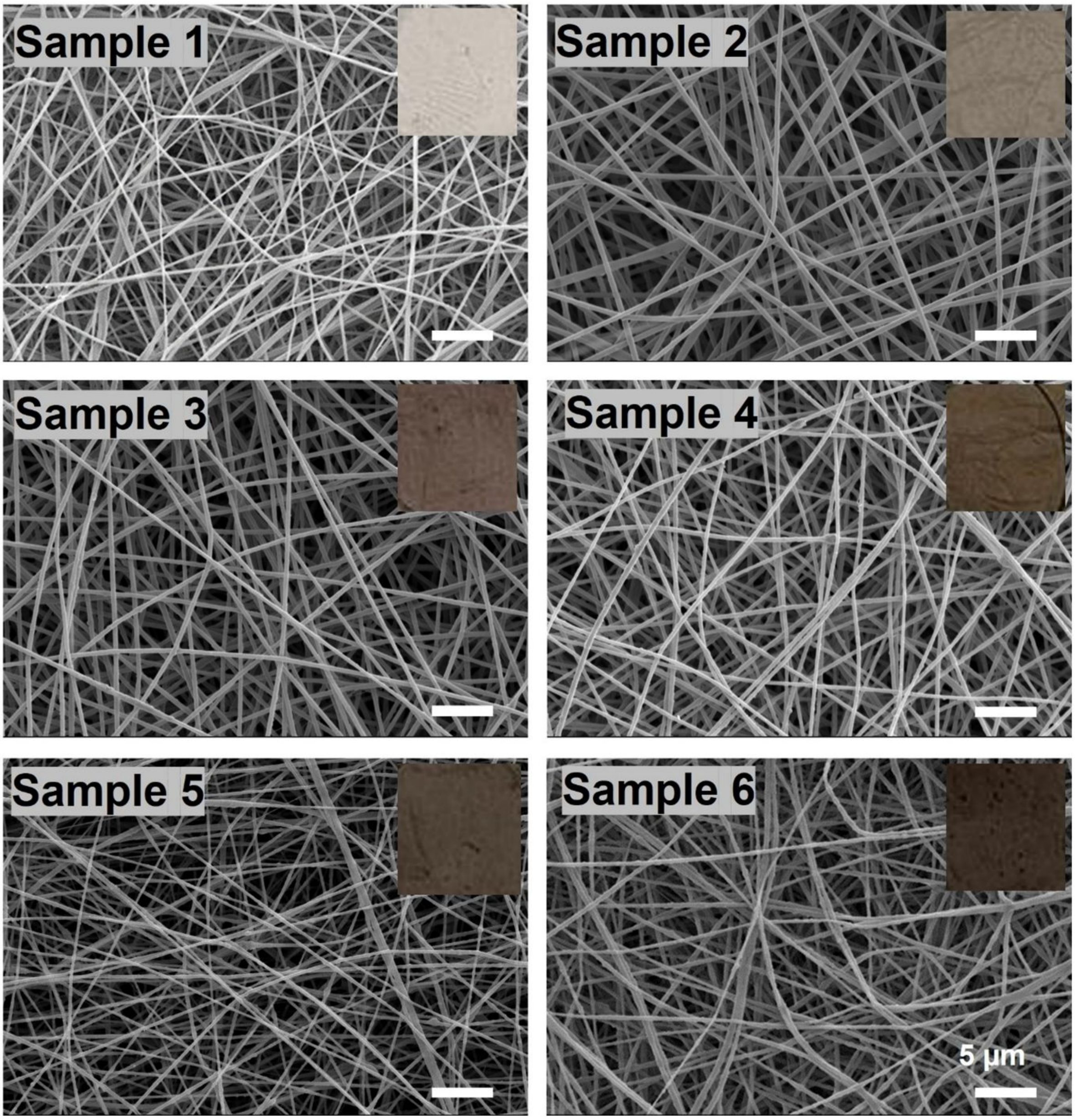

Figure S11. Photographs of as-spun nanofibrous mats listed in Table S1.

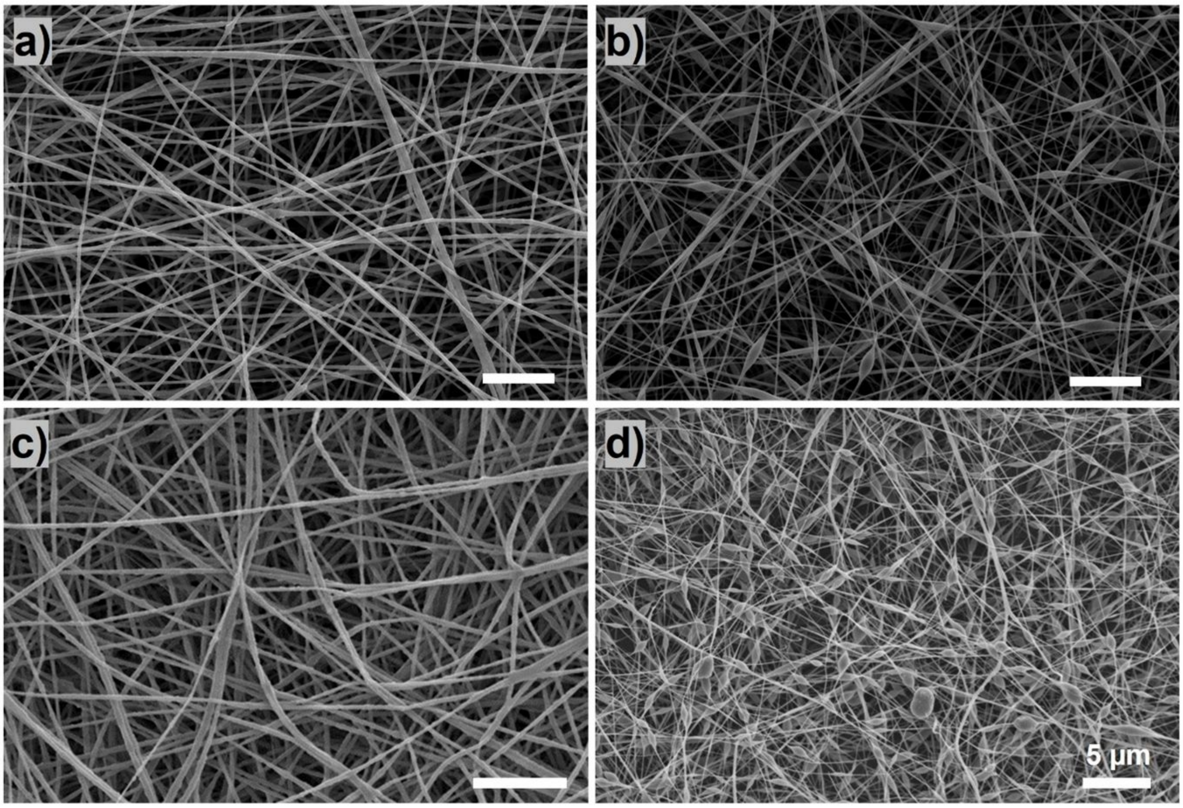

Figure S12. Comparison of electrospinning with and without MNPs: a) sample 5 with MNPs , b) sample 5 without MNPs , c) sample 6 with MNPs , and d) sample 6 without MNPs.

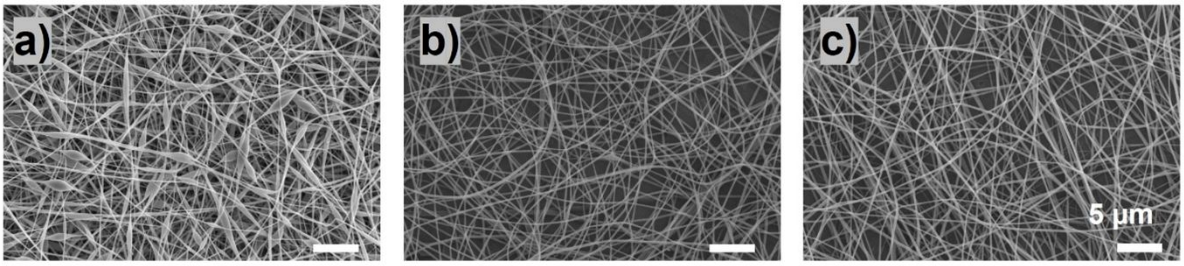

Figure S13. Optimization of electrospinning parameters for the PVA/PEO control (sample 5 formulation without MNPs): a) 16 kV, 400 µL/h; b) 16 kV, 200 µL/h; and c) 18 kV, 200 µL/h.

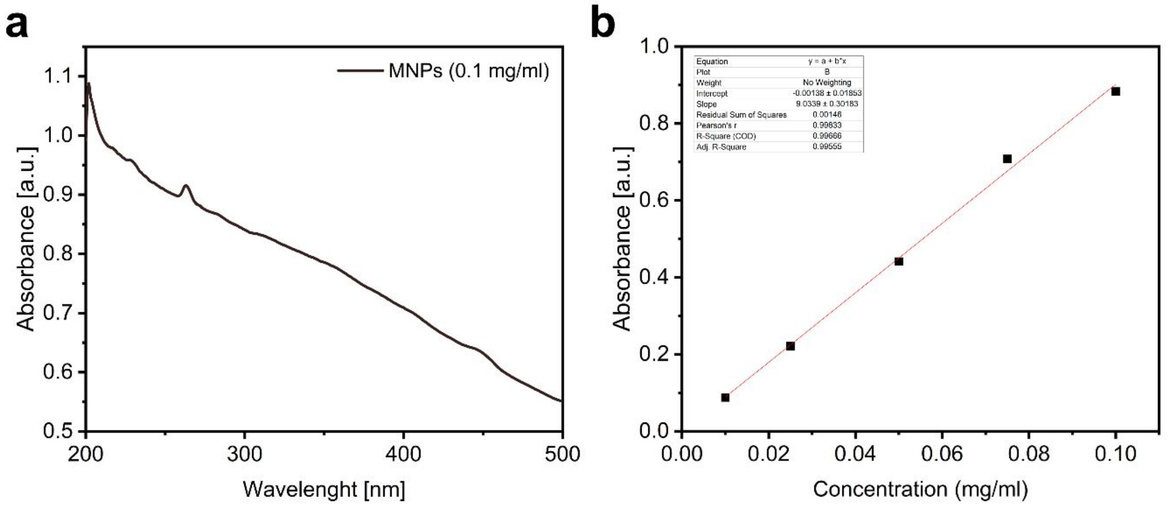

Figure S14. a) UV-VIS of melanin in PBS. b) Calibration curve of MNPs in PBS.
